# Quantification of protein isoforms in mesenchymal stem cells by reductive dimethylation of lysines in intact proteins

**DOI:** 10.1002/pmic.201100308

**Published:** 2012-01-13

**Authors:** Yi-Min She, Michael Rosu-Myles, Lisa Walrond, Terry D Cyr

**Affiliations:** Centre for Vaccine Evaluation, Biologics and Genetic Therapies Directorate, Health CanadaOttawa, Ontario, Canada

**Keywords:** Isotopic labeling, Mesenchymal stem cells, MS, Protein isoforms, Protein quantification, Technology

## Abstract

Mass spectrometry (MS)-based quantification of highly homologous proteins in complex samples has proven difficult due to subtle sequence variations and the wide dynamic range of protein isoforms present. Herein, we report the use of reductive dimethylation on intact proteins to quantitatively compare protein isoform expression in the nucleus and cytoplasm of mesenchymal stem cells (MSC) and normal stroma. By coupling fixed-charge MS/MS scanning, high-resolution UPLC FT-MS data-dependent acquisition and MASCOT-based data mining, hydrogen/deuterium-labeled dimethyl-lysine peptides were simultaneously captured allowing the accurate comparison of 123 protein isoforms in parallel LC MS/MS runs. Thirty-four isoforms were identified that had expression levels specific to MSC. Where possible, proteomic analyses were verified by Western blotting and were demonstrated to be divergent from the level of gene transcription detected for certain proteins. Our analysis provides a protein isoform signature specific to MSC and demonstrates the suitability of dimethyl-lysine labeling on intact proteins for quantifying highly homologous proteins on a proteome-wide scale.

## 1 Introduction

Isoforms are highly homologous proteins derived from gene polymorphism (SNPs) and alternative splicing of mRNA during transcription 1–3. The subtle variations in structure that arise among isoforms can result in functionally distinctive proteins from the same family. As such, the functions of complex biological systems, such as cells and bodily fluids, are often regulated by dynamic changes in the type and quantity of isoforms present. Thus, the ability to quantify differences in the expression of highly homologous proteins via proteome-wide analysis represents an important advancement for understanding the function of biological systems in both normal and diseased states.

In the past 10 years mesenchymal stem cells (MSC) have emerged as a biological system with tremendous therapeutic potential 4. Both pre-clinical and clinical studies have demonstrated the capacity of MSC containing stromal cultures to successfully treat diseases and tissue damage due to heart attack 5, diabetes 6, peripheral ischemia 7 and severe sepsis 8 among others. Despite this promise, our understanding of the proteins involved in the mediating MSC function has been limited. This has been due largely to an inability to reliably differentiate between MSC and non-stem cell stroma either in their resident tissues or in therapeutically relevant stromal cultures. Recent work from our group determined that stromal cell cultures derived from C57Bl/6 bone marrow (BM) provide a unique system for discriminating MSC and non-stem cell stroma through expression of the cell surface protein CD105 9. This culture system was shown to allow direct comparison of functional differences between MSC and normal stroma, thus providing a reliable means to identify a protein isoform signature specific to MSC.

MS-based detection methods have shown promise for accurate identification of protein isoforms in simple systems isolated by immunoaffinity purification or by 2D gel 10–12; however, high-throughput analysis of closely-related proteins has been challenging because of high sequence homologue and wide dynamic range of protein abundances 13. Bottom-up approaches have shown limited success since only a portion of the entire protein is generally detected at the peptide level and peptides common to all isoforms are of high intensities, rendering highly homologous proteins indistinguishable 14. Alternative top-down sequencing of intact proteins is more amenable to isoform quantification, however, the difficulty to dissociate large molecular mass proteins as well as limited sensitivity restrict its proteomic applications on a large scale 14. Thus, the employment of fractionation methods and alternative approaches to protein labeling and data analysis may be necessary to accomplish accurate quantification of protein isoforms from cell extracts and other complex samples 15.

Efficient and stable labeling of protein samples is a critical component of MS-based quantitative proteomics. Chemical labeling provides a universally suitable method for quantitatively comparing proteins from complex biological systems. Reductive dimethylation using formaldehyde and sodium cyanoborohydride is one of the most efficient methods used for quantitative proteomics 16–32. Several advantages of dimethyl labeling have been recognized in the earlier studies, including (i) low relative cost; (ii) fast reaction rate; (iii) high specificity to amino groups; (iv) mild reaction conditions; (v) high labeling efficiency; (vi) stable derivative products; (vii) high ionization efficiency; and (viii) low isotope effects of dimethyl peptides on the reversed-phase (RP) HPLC separation 16, 17, 19, 21, 31. Dimethyl labeling of peptides has been widely utilized for protein quantification 16–32; however, the labeling of intact proteins was only investigated on a restricted group of protein standards 30–32, and no systematic evaluation of the quantitative method on complex mixtures has been reported previously. A known constraint causing the difficulty of such analyses is that preferred proteases, trypsin and Lys-C, do not cleave the modified lysine residues 27. Analysis of complex samples using reductive dimethylation labeling has also been difficult since differentially labeled peptide pairs are not always detected by simultaneous MS/MS scanning resulting in unreliable quantitative ratios.

In this study, we have evaluated the suitability of reductive dimethylation labeling of intact proteins as a method to quantitatively compare highly homologous proteins in MSC and normal stroma. We have addressed several technical issues related to protein quantification using this protocol, and sought reasonable solutions to successfully compare the expression of 123 different protein isoforms in the both nuclear and cytoplasmic cell extracts. This work identifies protein isoforms expressed at unique levels in MSC and demonstrates the feasibility of highly homologous protein quantification through stable isotope labeling of intact proteins by reductive dimethylation.

## 2 Materials and methods

### 2.1 Proteins and chemical reagents

The following chemicals and protein standards were purchased from Sigma-Aldrich: bovine ubiquitin, bovine cytochrome C, bovine α lactalbumin, chicken lysozyme, horse myoglobin, bovine β lactoglobulin B, bovine milk casein, bovine α-acid glycoprotein, bovine carbonic anhydrase, chicken ovalbumin, BSA, bovine apotransferrin, triethanolamine (TEA), triethylammonium bicarbonate (TEAB), guanidine hydrochloride, ammonium bicarbonate (NH_4_HCO_3_), DTT, iodoacetamide, paraformaldehyde (CH_2_O), paraformaldehyde-d2 (CD_2_O, 98%), sodium cyanoborohydride (NaBH_3_CN), sodium cyanoborodeuteride (NaBD_3_CN, 96%), ammonium, and formic acid (FA). Deuterium oxide (D_2_O, 99.9%) was obtained from Cambridge Isotope Laboratories. Sequence-grade bovine trypsin was purchased from Roche Diagnostics.

### 2.2 Cell culture and protein isolation

MSC- and stem cell-depleted stroma cultures were derived from 8–12 week old C57B6/J mouse bone marrow by the methods previously described 9. Briefly, plastic adherent marrow cells were expanded in murine MesenCult complete medium for 4–8 wk prior to removal of hematopoietic and endothelial cells using a custom EasySep protocol (Stem Cell Technologies). The resulting pure stromal cell cultures were stained with phycoerythrin (PE) conjugated anti-CD105 (MJ7/18; eBiosciences) and CD105 expressing (CD105^+^) and non-expressing (CD105^−^) populations were isolated by fluorescence activated cell sorting (FACS). MSC function was measured in purified populations using the Murine Mesenchymal Stem Cell Detection Kit™ (R&D Systems). For nuclear and cytoplasmic protein subfractionation, ≥10×10^6^ CD105^+^ and CD105^−^ cell populations of the same culture age were collected, lysed in an ice-cold Dounce homogenizer and centrifuged at 10 000×*g* for 30 min to pellet intact nuclei. Nuclear and cytosolic protein fractions were isolated using the Nuclear/Cytosol Fractionation Kit from BioVision.

### 2.3 Reductive dimethylation of intact proteins and in-solution trypsin digestion

Proteins were modified by reductive dimethylation as described previously for peptides 27 and proteins 32. Bovine apotransferrin (100 μg) or a 12 protein mixture (200 μg) was dissolved in 100 mM NH_4_HCO_3_, reduced with 10 mM DTT and incubated in 55 mM iodoacetamide. Small molecules were removed by centrifugation using Amicon Ultra 3 kDa MWCO cut-off filter devices (Millipore) with buffer exchanges of 100 mM TEAB (pH 8.5) or 300 mM TEA/6 mM guanidine hydrochloride/20% methanol. Subsequent experiments were handled in a fume hood with CAUTION. In situ generation of 4% formaldehyde was freshly prepared by dissolving paraformaldehyde in H_2_O or paraformaldehyde-d2 in D_2_O at 59°C following addition of 1 μL of 0.1 M NaOH. Equal volumes of 0.6 M NaCNBH_3_ (light, L) or NaCNBD_3_ (heavy, H) and 4% CH_2_O or 4% CD_2_O were added in sequence and protein solution was incubated on an Eppendorf Thermomixer at room temperature for 2 h. After quenching with 4% ammonia, light and heavy labeled proteins were combined and excess chemical reagents removed by a centrifugal filtration. Protein mixtures were digested at 37°C overnight with 1:100 w/w sequencing-grade trypsin.

### 2.4 SDS-PAGE separation, Western blotting and in-gel tryptic digestion

Nuclear and cytosolic proteins were dimethylated, and separated by 10% SDS-PAGE gel (Bio-Rad Laboratories). Proteins were stained with Coomassie Brilliant Blue R-250, and the gel bands were excised. In-gel tryptic digestion was performed using 50 ng of trypsin in 25 mM NH_4_HCO_3_ at 37°C overnight. The peptides were extracted twice with 0.1% trifluoroacetic acid (TFA) and 60% ACN/0.1%TFA, dried on a SpeedVac (Savant), and reconstituted in 0.1% FA for LC MS/MS analysis. For Western blotting, equal amounts of protein from CD105^+^ or CD105^−^ cells was separated on 12% acrylamide gels and transferred to PVDF membranes. Western blots were incubated with rabbit anti-mouse polyclonal Hmgb2 (Abcam) and Septin6 (Protein Tech Group), rabbit monoclonal anti-mouse Hmgb3 (Epitomics, Clone ID EP2839Y) and goat anti-mouse polyclonal Septin7 (AbD Serotec). Proteins were visualized using either ECL Anti-Rabbit IgG HRP (GE Healthcare) or anti-Goat IgG-HRP (Santa Cruz Biotechnology).

### 2.5 UPLC LTQ-FT MS/MS analysis

Online LC MS/MS analysis was performed on a Nano-Acquity ultra-performance liquid chromatography system (UPLC, Waters) coupled to a 7-tesla hybrid linear ion trap Fourier transform ion cyclotron resonance mass spectrometer (LTQ-FT ICR, Thermo Fisher). The peptides were trapped by an RP Symmetry C18 column (180 μm id×20 mm length, 5 μm) at 5 μL/min, and subsequently separated on a C18 analytical column (100 μm id×100 mm, 1.7 μm, BEH 130) at 500 nL/min. Peptides were eluted using a mobile phase consisting of solvent A (0.1% FA) and solvent B (97.9% ACN/0.1% FA /2% water). NanoUPLC separation was achieved by a linear gradient from 5 to 45%, and then 85% of solvent B at a duration of 90 min for the digest of protein standards and gel isolates, or 4 h for the digest of the more complex subcellular fractions. To maximize recovery of samples for LC analyses, the limited amounts of in-gel digests were loaded into Waters total-recovery vials (P/N 186000384c) and the needle level of UPLC sample injection was near the bottom of a vial (set to 0.5 mm height).

Two instrumentation methods were used for MS/MS data acquisition by an LTQ FT Ultra 2.5.5 and Xcalibur 2.0.7 software. MS/MS measurements were typically conducted in the data-dependent mode following a full FT-MS survey scan over a mass range of m/z 300–2000. Survey scans were acquired in the ICR cell with a resolution of 100 000. Multiply charged peptide ions (2+, 3+ and 4+) were isolated for MS/MS analysis of the top eight most intense precursor ions by the LTQ. The target values of automatic gain controls (AGC) were 1 000 000 for FT-MS and 10 000 for the LTQ MS/MS. Ion fragmentation was achieved with the helium gas at a normalized collision energy of 35%. Fixed charge scanning of doubly, triply or quadruply charged ions was selected for each data-dependent MS/MS acquisition of complex peptide mixtures. Dynamic exclusion was enabled for a period of 180 S. A subgroup of peptide ions with the same charge state were analyzed in parallel LC MS/MS runs to allow a high probability of simultaneous detection of isotopically labeled dimethyl peptide pairs.

### 2.6 Protein identification and quantitation

Protein identification was performed using an in-house MASCOT Server (version 2.3.0, Matrix Science), and the data were searched against the Swiss-Prot–UniProt database for the standard proteins, or the National Center for Biotechnology Information (NCBInr) mouse database (downloaded on January 15th, 2010, 10098342 sequences) for cell extracts. The parameter setting of trypsin digestion allowed for four missed cleavage sites. Dimethyl peptide pairs were identified using two fixed modifications of the light labeling of lysines together with the carbamidomethylation of cysteine residues, variable deamidation modifications of asparagine and glutamine, methionine oxidation and heavy labeling dimethylation of lysines. Mass tolerances were set to 10 ppm for the FT MS ions and 1 Da for ion trap MS/MS fragment ions. Peptide assignments were filtered by an ion score cut-off of 20, if necessary, the significance threshold was adjusted to 0.01 to achieve a false discovery rate of <3%.

Protein quantification was processed by the MASCOT Distiller software (version 2.3.2.0). The raw FT MS data were centroided at a peak half-width of 0.025 and 400 points/Da. The maximum ion charge state was set to 5, and the Sum method was used as the scan group aggregation. MS/MS processing was centroided at a peak half-width of 0.2 and 20 points/Da, and regridded with the same value. Time domain was used as the scan group aggregation at the precursor mass range of 300–16 000 Da. The MS peak picking was accomplished with 500 iterations, and filtered through a correlation threshold of 0.5, minimum signal-to-noise ratio of 2, and *m/z* range from 50 to 100 000. FT-MS peak profile was determined at a minimum width of 0.001 Da and maximum peak width of 1 Da.

The identified peptides from MASCOT MS/MS search were directly imported into MASCOT Distiller to generate a quantification report of heavy/light (*H/L*) ratios. The output of *H/L* ratios was thus limited to those dimethylated peptides that were matched with the confident sequence identification and the identical charge state. Precursor ion protocol was used for peptide quantification, and the ratios were calculated using the peak areas of extracted ion chromatograms (XICs) based on the trapezium integration method. To improve the accuracy of the results, the impurity correction of deuterated reagents (98% CD_2_O and 96% NaBD_3_CN) was incorporated into the quantification method. Manual inspection of the protein isoforms was conducted on the dimethyl lysine-containing peptides based on the valid sequence identification by MASCOT search. The *H/L* ratios were calculated by the integrated XIC peak area of isotopic peptide ions and manually verified in each case.

### 2.7 Real-time reverse transcriptase PCR

Total-RNA was extracted using the RNeasy Plus Mini Kit (Qiagen) according to the manufacturer's protocol. The RNA samples were treated with DNASE (TURBO DNA-free kit, Applied Biosystems) to remove contaminating DNA and quantified on a spectrophotometer (Nanodrop). cDNA was synthesized from 2 μg of RNA using the Superscript III First Strand cDNA Synthesis System for RT-PCR (Invitrogen). Real-time PCR was performed using the Power Sybr Green PCR Master Mix and the samples were run on the 7500 Fast Real-Time PCR System (Applied Biosystems). Quantitative comparisons were made using the delta/delta Ct method by first measuring the expression of each gene in CD105^+^ and CD105^−^ cell-derived cDNA relative to the expression level of actin. The ratio of relative expression in CD105^+^/CD105^−^ cells was then determined.

## 3 Results and discussion

### 3.1 Application of reductive dimethylation of intact proteins for quantification of proteins in cell extracts

To demonstrate the feasibility of using reductive dimethylation of intact proteins to quantify proteins in complex mixtures, we first tested the labeling efficiency and reliability of the method on a model protein, bovine apotransferrin (78 kDa). Heavy and light labeled proteins were combined and digested with trypsin to create dimethyl peptide pairs with 6 Da mass differences per lysine residue (Supporting Information Fig. S1). These doublet mass spectral peaks and extracted ion chromatograms served as the basis for protein quantification. The extent of chemical modifications was examined by mixing an equal ratio of the heavy and light labeled proteins followed by LC LTQ FT-MS analysis on the tryptic digest. Of the 23 high-abundance peaks inspected in the total ion chromatogram (TIC) of apotransferrin (Supporting Information Fig. S2a), 19 of them contained 22 dimethylated peptide pairs (Supporting Information Table S1,) and the remaining 4 were lysine-free peptides and, therefore, had no label. The MASCOT database search identified peptides which account for 93% of the sequence bovine apotransferrin (Swiss-Prot–UniProt, Q29443). The dimethylation reaction exhibited a high specificity for lysine residues, and by products of cyanide-induced side reactions, which have been reported to compete with reductive dimethylation 33, 34, were not detected in any of our experiments. In addition, neither hydrogen/deuterium post-exchange at the modified sites nor back-labeling at the N-termini of the peptides was found to occur during the sample preparation. Peaks corresponding to peptides cleaved at unmodified lysine residues (Supporting Information Table S1) were detectable, but appeared at intensities of <5% and their *H/L* ratios matched to those of the high-abundance dimethyl peptides. Thus, our results show dimethyl labeling efficiency of over 95%, which is consistent with the previously reported nearly complete reactions for small intact proteins (myoglobin and RNase A) 31. Additional examination was conducted on a second data set of 55 high-intensity TIC peaks from a mixture of 12 protein standards (Supporting Information Fig. S2b and Table S2). A total of 60 peptides were identified, in which 41 dimethylated peptides and 15 non-lysine-containing peptides (without any label) exhibited cleavages specifically at arginine. The other four C-terminal-lysine ending peptides were also dimethylated, and three of which were cleaved at the unexpected site of dimethylated lysine at a very low intensity (<4%) (Supporting Information Fig. S3 and Table S2). Not surprisingly, all lysine-containing peptides were modified by dimethylation. The high efficient labeling of intact proteins by reductive dimethylation could be partially attributed to the small size of the chemical reagents (formaldehyde, sodium cyanoborohydride) which are able to penetrate into the interior of protein structure. Steric hindrance is minimized by protein denaturing and alkylation of cysteines before the labeling, resulting in a full coverage of all lysine sites.

**Figure 1:**
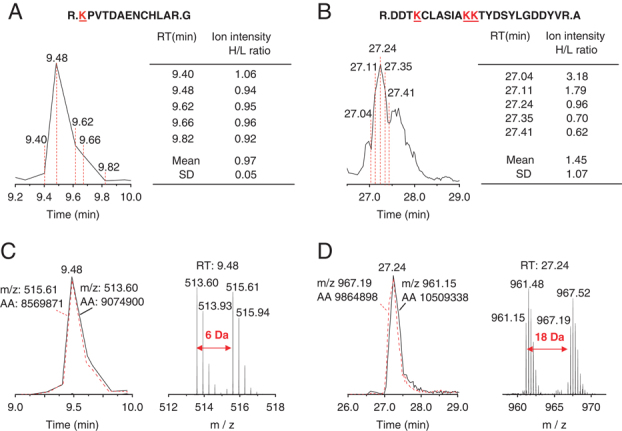
Isotope effect of dimethyl peptide pairs on the RP-LC elution. (A) Expanded view of the TIC peak at 9.48 min for quantitative analysis of a single lysine-containing peptide. The calculated H/L values of the mass peak intensities between the triply-charged dimethyl peptide ions of *m/z* 513.60 and *m/z* 515.61 are shown at the different elution times. (B) The XICs of the ions at *m/z* 513.60 (solid line) and *m/z* 515.61 (dash line). (C) Expanded view of the peak of a triple lysine-containing peptide at 27.24 min. The H/L ratios of the peak intensities between the triply charged dimethyl peptide ions of *m/z* 961.15 and *m/z* 967.19 at various time points are listed in the table. (D) The profile of XICs at *m/z* 961.15 (solid line) and *m/z* 967.19 (dash line). AA refers to the peak area.

Measurements of relative peak intensities for isotopically labeled peptide pairs in a single MS data set and the peak areas of XICs for precursors are widely used approaches for quantitative analysis of proteins. However, the determination of peptide ratios using MS peak intensities may be unreliable due to the shift of elution time between the light and heavy labeled counterparts on the RP-LC separation 24, 26, 27. This isotope effect of co-eluted peptides becomes significant with increasing number of deuterium atoms. Since the dimethylation labeling of peptides occurs at amino groups of both the peptide N-terminus and lysine residues, deuterium effect is expected to be minimized by protein labeling exclusively on the lysine residues prior to digestion and separation of peptides using high-resolution UPLC. Fig. 1A and C demonstrates this for the simplest case of a single lysine-containing peptide, no obvious isotope effect on the *H/L* ratios (0.97±0.05) was observed. Nevertheless, a slight shift in peak retention times (RTs) between the light and heavy pairs of multiple lysine-containing peptides was detected (Fig. 1B), yielding differences of the *H/L* ratios ranging from 0.62 to 3.18 at various time points. This data is consistent with the observations on the dimethyl labeling of individual amino acids reported by Guo et al. 24. As such variations could result in unacceptable errors during protein quantification, the integration of peak areas for entire XICs was utilized to obtain an accurate *H/L* ratio of the protein. Analysis of multi-lysine-containing apotransferrin peptides via this method determined an *H/L* ratio of 0.94 (9864898/10509338) (Fig. 1D), which is in agreement with the expected value of the protein mixture. Consequently, UPLC separation together with integration of peak areas through an XIC-based data processing provides an integrated approach for quantitative comparison of deuterated and non-deuterated peptides.

To explore the linearity of our quantitative method, light and heavy dimethylated bovine transferrin were mixed at *H/L* ratios of 1:1, 2:1, 4:1, 6:1, 8:1, 10:1, and the tryptic digests were analyzed in triplicate under the identical LC MS/MS conditions. As shown in Fig. S4a (Supporting Information), linear regression analysis revealed a linear relationship (*y*=0.9809*x*−0.1040, *R*^2^=0.9878) between the measured and expected *H/L* ratios. Similar analysis was further reproduced on a mixture of 12 protein standards (ubiqutin, cytochrome C, α-lactalbumin, lysozyme, myoglobin, β-lactoglobulin B, β-casein, α-acid glycoprotein, carbonic anhydrase, ovalbumin, BSA, apo-transferrin). Analyses of these proteins in triplicate, at the *H/L* ratios of 1:1, 1:2, 2:1, 3:1 and 1:3, revealed reproducible results for two sets of independent experiments using different buffer solutions. The accuracy of the protein ratios was determined with an average error of 3.25% (buffer A: 300 mM TEA/6 mM guanidine hydrochloride/20% methanol) (Supporting Information Fig. S4b) or −4.56% (buffer B: 100 mM TEAB). Thus, through analysis of protein standards we have demonstrated that dimethylation of intact proteins is an efficient labeling method that allows reliable and linear quantification of peptides using LC MS/MS.

As with all high-throughput proteomics methods, quantification using dimethyl labeling of intact proteins is more difficult when analyzing complex samples. Accurate quantification of proteins requires reliable identification of labeled peptide pairs by MS/MS at the identical charge states. LC MS/MS scanning by data-dependent acquisition (DDA) is normally performed for top eight high-abundance ions following a single MS survey scan, in which all multiple charged ions are selected. The presence of peptides that do not contain lysine residues, usually at high intensities (Supporting Information Tables S1 and S2), can interfere with the reliable detection of *H/L* ratios for heavy and light labeled peptide pairs due to missing detection or unmatched charge states of them. To mitigate this limitation, we explored an instrumental fractionation method involving fixed charge MS/MS scanning on each of the multiply charged ions (2+, 3+, 4+ and up) to increase the probability of detecting the labeled peptides. From these, a subgroup of peptide ions with the same charge state (e.g. 2+ or 3+) were selected for analyses in parallel LC MS/MS runs, and the data were combined.

To explore the utility of the fixed charge MS/MS scanning method we quantitatively compared extracts from MSC enriched, (CD105^+^) and MSC-depleted (CD105^−^), stromal cells. Sample complexity was further reduced by subfractionation of nuclear and cytosol-specific protein extracts prior to dimethyl labeling. Labeling was detected at an efficiency of 95% or greater as demonstrated with apotransferrin standards. As shown in Table S4 (Supporting Information), our method allowed detection of a complete set of light and heavy labeled peptides, using two parallel LC MS/MS scanning experiments of the doubly and triply charged ions. These data demonstrate that charge-based fractionation of peptide ions offers an alternative approach for validating the sequence identification of both labeled dimethyl peptide pairs, and thus enhances the reliability of protein quantification in a complex biological sample. In addition, the list of total proteins identified from nuclear and cytoplasmic subfractions was analyzed using Ingenuity Pathway Analysis (IPA) software to estimate the purity of our fractionation technique. The top networks identified from nuclear and cytoplasmic extracts of both CD105^+^ and CD105^−^ cells were highly enriched for proteins that were properly assigned to the subfraction from which they were derived (Supporting Information Fig. S5). Overall, we have shown that dimethylation of intact proteins can be utilized in an efficient and accurate manner to compare the quantity of proteins in both a standard mixture and complex samples of cell extracts.

### 3.2 Differential analysis of protein isoform specific dimethylated peptides in CD105^+^ and CD105^−^ stroma

One of the challenges in quantifying differences among protein isoforms in a proteome-wide scale is the missed detection of isoform-specific peptides due to masking from more abundant isoform-common peptides. We hypothesized that, as dimethylation of lysine residues restricts trypsin cleavage sites to arginine, the generation of larger peptide sequences would afford a greater opportunity for detection of unique isoforms. To test this, we analyzed the capacity of reductive dimethylation of intact proteins to quantify highly abundant tropomyosin (TPM) isoforms in MSC-enriched CD105^+^ cells and MSC-depleted CD105^−^ cells. The TPM family consists of four isoforms: TPM1; TPM2; TPM3 and TPM4 that exhibit 73–83% sequence homology. Variable residues within these proteins are localized at sequence regions near the N-terminus and C-terminus (Supporting Information Fig. S6). To determine whether TPM isoform-specific peptides were uniquely identified through dimethyl labeling prior to digestion, we compared our results to those from LC MS/MS scans on tryptic digests of non-labelled CD105^+^ nuclear proteins. In the absence of dimethyl labeling, only peptides specific to TPM1, 3 unique peptides (3U), TPM3 (2U) and TPM4 (9U), but not TPM2, could be identified (Supporting Information Table S3). More specifically, dimethyl labeling and subsequent analysis of 2+ and 3+ charged ions identified 11 dimethylated peptide pairs that were unique to the individual TPM isoforms (Supporting Information Table S4) consisting of 3, 2, 2 and 4 peptides were specific to TPM1, TPM2, TPM3 and TPM4, respectively. Determination of longer peptide ions at 4+ charge state extended the protein sequence region (Supporting Information Table S5), allowing more isoform-specific peptides to be detected. For all charge states, this resulted in a total of TPM1(7U), TPM2(8U), TPM3(7U), and TPM4(9U) unique dimethyl-labeled peptides to be identified. Taken together, our analysis demonstrates that dimethyl-labeling of intact protein prior to tryptic digest allows better resolution and identification of protein isoforms.

**Figure 2:**
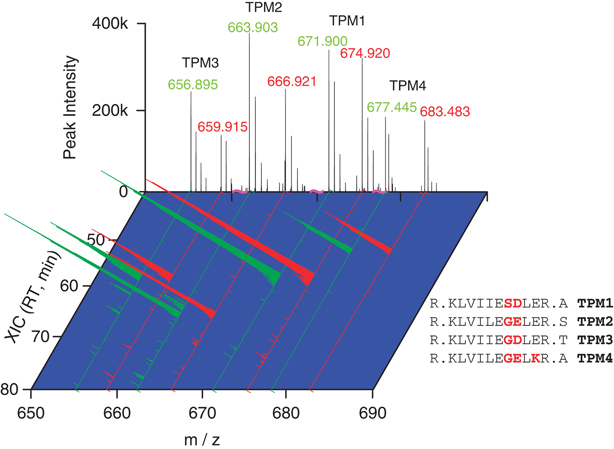
The 3D view of the doubly-charge peptide ions of tropomyosin (TPM) isoforms by reductive dimethylation in terms of intensity, *m/z* and XICs. Cytosolic proteins from MSC and stroma were dimethylated at lysine residues with light and heavy labels. The labeled samples were mixed and analyzed by LC MS/MS. Light and heavy labeled peptides are illustrated in green and red, respectively.

Manual inspection of the peak areas of XICs revealed consistent *H/L* ratios between the nuclear and cytosolic extracts from either cell type (Table 1). There was also very little variability detected among the *H/L* ratios of protein specific peptide fragments and of equivalent peptide ions at different charge states (SD <0.1 in all cases). Both TPM1 and TPM4 retained similar protein expression levels (*H/L* ∼1:1), whereas a subtle difference was determined in TPM2 and TPM3 which were both slightly down-regulated (*H/L*, 0.72) in the CD105^+^ stem cells (Fig. 2). These changes were not related to the degree of sequence homology between proteins (Supporting Information Fig. S6a). Several short non-lysine-containing peptides were also observed (Supporting Information Table S4), most of which contained sequences conserved between isoforms. While comparing to that obtained from DDA scan of the top eight ions, the fixed-charge scans increased the number of MS/MS measured peptide ions by a range of 114–280% which also covered both light and heavy labeled peptides at the identical charge states (Supporting Information Table S6). These data demonstrate that LC MS/MS analysis of peptides generated from dimethyl labeling of intact peptides provides a reliable method for comparing the quantity of individual protein isoforms among MSC and non-stem cell-containing stroma.

**Table 1:** Quantitative measurements of tropomyosin isoforms by reductive dimethylation

Protein	Peptide	Sequence	Ion pairs *m/z* (charge)	*H/L* (nucl.)	*H/L* (cyto.)
TPM1	22–35	R.AEQAEADKKAAEDR.S	794.4022(2+); 800.4391(2+)	–; –	0.96; –
			529.9372(3+); 533.9625(3+)	0.98; 1.02	0.92; 0.93
	126–133	R.GMKVIESR.A	474.2701(2+); 477.2896(2+)	0.93; 0.98	0.95; 0.92
			316.5162(3+); 318.5285(3+)	1.12; 0.91	0.99; 1.00
	168–178	R.KLVIIESDLER.A	671.8997(2+); 674.9197(2+)	0.93; 0.91	0.90; 0.98
			448.2692(3+); 450.2819(3+)	1.07; 0.97	0.91; 0.95
			Mean±SD	0.98±0.07	0.95±0.03
TPM2	22–35	R.AEQAEADKKQAEDR.C	822.9127(2+); 828.9512(2+)	–; –	0.65; 0.68
			548.9445(3+); 552.9698(3+)	0.68; 0.75	0.67; 0.72
	168–178	R.KLVILEGELER.S	663.9026(2+); 666.9211(2+)	0.69; 0.73	0.71; 0.77
			442.9382(3+); 444.9502(3+)	0.76; 0.73	0.72; 0.74
			Mean±SD	0.72±0.03	0.71±0.04
TPM3	13–27	R.KIQVLQQQADDAEER.A	899.9688(2+); 902.9877(2+)	0.77; 0.67	0.70; 0.75
			600.3138(3+); 602.3276(3+)	0.76; 0.78	0.72; 0.75
	132–142	R.KLVIIEGDLER.T	656.8951(2+); 659.9147(2+)	0.78; 0.60	0.65; 0.58
			438.2652(3+); 440.2782(3+)	0.69; 0.71	0.65; 0.82
			Mean±SD	0.72±0.06	0.70±0.07
TPM4	13–27	R.KIQALQQQADDAEDR.A	878.9446(2+); 881.9642(2+)	1.13; 1.24	1.20; 1.21
			586.2992(3+); 588.3115(3+)	1.10; 1.11	1.19; 1.20
	43–54	R.EKAEGDAAALNR.R	636.8301(2+); 639.8492(2+)	1.17; –	1.17; 1.17
			424.8895(3+); 426.9019(3+)	1.07; 1.08	1.24; 1.21
	66–89	R.AQEQLATALQNLEEAEKA	1336.6539(2+); 1339.6765(2+)	–; –	1.17; 1.15
		ADESER.G	891.4391(3+); 893.4516(3+)	1.22; 1.24	1.20; 1.18
	132–142	R.KLVILEGELKR.A	677.4452(2+); 683.4827(2+)	0.96; 1.04	1.06; 1.08
			451.9652(3+); 455.9905(3+)	0.98; 1.04	1.08; 1.05
			Mean±SD	1.11±0.09	1.16±0.06

LC MS/MS analysis was performed in duplicate at a duration of 240 min, and *H/L* ratio were calculated based on manual inspection of the peak area of XICs with the correction of isotopic deuterium contents in the starting materials (98% paraformaldehyde-d2; 96% NaBD_3_CN). “–” represents the *H/L* ratio unavailable due to low-intensity peaks. SD: standard deviation.

Similar comparison was performed on the dimethyl peptides of several high abundance protein isoforms (Supporting Information Fig. S6b), and the isoform-specific peptides were well-resolved by LC MS/MS (Supporting Information Fig. S7). The details of quantitative results are shown in Table 2 and Table S7 (Supporting Information), in which the sequence identities of protein isoforms were shown in Table S8 (Supporting Information). These included cytoskeletal and house-keeping proteins that were present at high abundance in the nuclear and cytosolic extracts, such as actin (α, β, γ), actinin (α 1 and 4), tubulins and myosin (light and heavy chains). As expected, CD105^+^ and CD105^−^ stroma showed no differences in the overall expression levels of isoforms for actin and actinin house-keeping proteins, as indicated by *H/L* ratios approaching one. In contrast, a decrease of at least twofold was identified in the amount of tubulin α 1A (*H/L*=0.51), 1B (*H/L*=0.51), β 2 (*H/L*=0.25) and 2C (*H/L*=0.57) isoforms in CD105^+^ cells. As tubulins are usually cell-type specific, our data suggest the possibility that distinct tubulin isoforms can further discriminate MSC from normal stroma.

**Table 2:** Abundance changes of protein isoforms between MSC and normal stroma

Accession #	Protein ID	*H/L* (nucl.)	*H/L* (cyto.)	Accession #	Protein ID	*H/L* (nucl.)	*H/L* (cyto.)
gi|71037403	Myosin, Lc, B	0.42	–	gi|74315975	Psmd1	–	2.80
gi|6755714	Transgelin 1	2.08	1.50	gi|19882201	Psmd2	–	2.05
gi|30519911	Transgelin 2	1.94	1.61	gi|19705424	Psmd3	–	2.00
gi|85060507	HnRNP, A1	2.00	–	gi|6754724	Psmd7	–	2.21
gi|13384620	HnRNP, K*	1.89	6.11	gi|72679790	Psmd11	–	2.14
gi|3329496	HnRNP, U*	1.02	4.72	gi|6755210	Psmd13	–	2.68
gi|6680229	Hmg B2	2.09	–	gi|6753320	CCT3, γ	–	3.50
gi|6680231	Hmg B3	2.14	–	gi|148666717	CCT7, eta	2.05	1.64
gi|53733821	Tubulin, α 1A	–	0.51	gi|5295992	CCT8, theta	1.60	1.91
gi|34740335	Tubulin, α1B	–	0.51	gi|6754816	Septin 2	3.62	–
gi|4507729	Tubulin, β 2	–	0.25	gi|5689158	Septin 6	2.28	–
gi|13542680	Tubulin, β 2C	–	0.57	gi|148693353	Septin 7	2.66	–
gi|124517663	Annexin A1	–	0.39	gi|122889413	Septin 9	1.97	–
gi|161016799	Annexin A4	–	0.36	gi|31542366	CDC2	–	1.92
gi|23956214	Sfpg	2.10	–	gi|60650308	LRR 27	0.71	0.52
gi|4507773	UBE2D 1	–	4.35	gi|13385296	Bzw1	–	3.01
gi|4507777	UBE2D 3	1.81	2.03	gi|254587996	Anp32e	–	7.64

*H/L* values are calculated from the average of two independent sample preparations, and 34 proteins with at least two-fold difference are listed (see Supporting Information Table S4). “–” represents weak or undetected peaks.

Of the high-abundance isoforms detected, the myosin light chains (Lc3, Lc4 and Lc6), were of particular interest as quantitative differences were detected between the nuclear and cytosolic subfractions of CD105^+^ and CD105^−^ cells. Specifically, the *H/L* ratio of each isoform was 0.59 in nuclear extracts and 1.33, 1.43, and 1.32, respectively, in the cytosol subfractions. Transgelin 1 and 2, which had nuclear *H/L* ratios of 2.08 and 1.94 and cytosolic ratios of 1.50 and 1.61 respectively, were the only other isoforms present at differing concentrations in the nucleus and cytoplasm of CD105^+^ cells compared with CD105^−^ cells. While the significance of these data is unclear, the results suggest that, when combined with cellular subfractionation techniques, reductive dimethylation of intact proteins may provide a novel method to monitor variations in subcellular location.

### 3.3 Isoforms associated with regulating cell proliferation, differentiation and apoptosis show distinct expression levels in CD105^+^ and CD105^−^ stroma

Stem cells are defined by a distinct signature of cell proliferation, differentiation and apoptotic resistance. Our analysis revealed that several protein isoforms related to processes of cell proliferation, differentiation and apoptosis were present at twofold greater or lower quantities in CD105^+^ cells. Proteins involved in cell proliferation and differentiation included transcription factors and heterogeneous nuclear ribonuclearprotein (hnRNP) family members. Of the eight hnRNP's detected, only A1 (2.0-fold), K (6.1-fold) and U (4.7-fold) isoforms were differentially expressed in CD105^+^ cells. Interestingly, hnRNP K and U isoforms were up-regulated in the cytosolic subfraction but not in the nucleus of CD105^+^ cells, suggesting that the ability of these proteins to maintain mRNA stability outside the nucleus may be an important function in MSC.

Transcription factors with altered expression in CD105^+^ cells included high mobility group box (Hmgb) isoforms 2 and 3 which were detected only in the nuclear fractions regardless of cell type. Comparison of *H/L* ratios determined that MSC enriched CD105^+^ cells contained 2.1-fold greater amounts of Hmgb2 and Hmgb3, compared with CD105^−^ cells. Hmgb family proteins have been implicated in embryonic development, cartilage formation, hematopoietic stem cell proliferation and regulating inflammatory responses. Our data imply that Hmgb2 and 3 may also play a role in regulating the proliferation, differentiation or immune regulatory functions of MSC.

Members of the annexin protein family have been reported to play a role in apoptosis, endocytosis, membrane organization and inflammatory regulation. Reductive dimethylation analysis identified seven annexin isoforms of which two, Anxa1 and Anxa4, were downregulated greater than twofold in CD105^+^ cells compared with CD105^−^. Further investigation into the function of annexins in MSC may determine the importance of Anxa1 and Anxa4 downregulation in these cells. Overall, our reductive dimethylation analysis demonstrates that quantitative differences in protein isoforms exist between MSC and non-stem cell stroma, and provides a basis for further study into proteins that regulate MSC function.

### 3.4 Quantitative comparison of low abundance isoforms in CD105^+^ and CD105^−^ stroma

To further reduce sample complexity and allow a more comprehensive assessment of low-abundance isoforms, dimethylated proteins were separated by molecular weight using SDS-PAGE gel electrophoresis. A total of 41 gel bands were excised from the nuclear and cytosolic extracts of CD105^+^ and CD105^−^ cells (Supporting Information Fig. S8) and subsequently analyzed by LC MS/MS following in-gel tryptic digestion. The MASCOT search of these data identified 1797 nuclear proteins and 1059 cytosolic proteins from the combined extracts, and resulted in the identification of 57 protein isoforms not identified in our initial analysis. Quantitative comparison revealed that 14 of these were differentially expressed at levels two-fold or greater in CD105^+^ cells compared with CD105^−^. As shown in Table 2, members of the ubiquitin-mediated protein degradation pathway comprised a large portion of the proteins identified as up-regulated in CD105^+^ cells. The ubiquitin-conjugating enzymes E2D (UBE2D) 1 and 3 were enriched 4.4- and 2.0-fold respectively in CD105^+^ cell cytoplasm. In addition, partial regulatory particles of the 26S proteasome involved in ubiquitin-mediated degradation (Psmd1, 2.8; Psmd2, 2.1; Psmd3, 2.0; Psmd, 2.21; Psmd11, 2.1; Psmd13, 2.7) were up-regulated exclusively in the cytoplasm. These alterations may reflect mechanisms of protein regulation within MSC through modification of ubiquitination and deubiquitinating activity.

### 3.5 Comparison of quantitative RT-PCR and reductive dimethylation methods reveals differences in the transcriptomic and proteomic content of MSC

**Figure 3:**
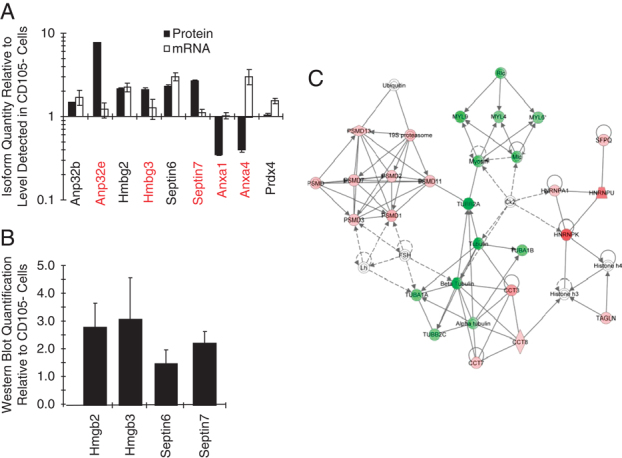
Comparison of reductive dimethylation, real-time RT-PCR and Western blot methods for quantification of growth and differentiation-associated genes and the identification of MSC-associated protein networks by IPA analysis. Cytosolic and nuclear protein fractions and total RNA were isolated from CD105^+^ and CD105− stroma. (A) Protein fractions were labeled with distinct hydrogen isotopes by reductive dimethylation while total RNA was utilized for cDNA synthesis and real-time PCR analysis for expression of Anp32, Sept, Hmg, Anxa and Prdx gene isoforms. Changes in quantity of protein isoforms expressed in CD105^+^ cells relative to CD105^−^ were determined by LC MS/MS, where values were calculated as a mean±SEM of intensities from at least two peaks (filled bars). Quantitative gene expression relative to CD105^−^ cells was determined as a mean±SEM from three separate samples (clear bars). (B) Total cell protein extracts were separated by SDS-PAGE and analyzed by Western blot using antibodies specific to Hmgb2, Hmgb3, Septin6 or Septin7. Isoform expression levels in CD105^+^ cells relative to CD105^−^ were determined by densitometry. Results were normalized based on comparative quantification of beta-actin in order to account for errors in protein loading. (C) Protein isoforms that were upregulated (red) or downregulated (green) in CD105^+^ cells were analyzed by IPA. The diagram depicts the only network identified with a score >50 in which α- and β-tubulin play a central role.

Traditionally, the investigation of molecules and pathways that are biologically important in complex systems has been completed using mRNA quantification techniques. However, the results of these assays may be reflective of changes in protein synthesis and not necessarily protein concentration. To determine the degree of correlation between protein and mRNA expression in MSC, we used real-time RT-PCR to quantitatively compare the total RNA extracts from CD105^+^ and CD105^−^ stroma. For this comparison we chose to analyze the expression of 10 different genes representing protein isoforms that were identified by LC MS/MS and may have a role in regulating MSC proliferation, differentiation or apoptosis. The results illustrated in Fig. 3A indicate changes in mRNA and protein isoform expression levels detected in CD105^+^ cells relative to CD105^−^ cells by real-time PCR (clear bars) and reductive dimethylation (filled bars), respectively. As outlined in red, the level of mRNA detected was significantly divergent from that of protein isoform levels in many of the genes analyzed. These included isoforms that were up-regulated at the protein level in CD105^+^ cells (Anp32e (7.64±0.01); Septin7 (2.66±0.08); Hmgb3 (2.14±0.09)) but showed no difference in mRNA expression (1.2±0.25; 1.1±0.1; 1.2±0.5, respectively). Protein isoforms that were detected at twofold lower levels in CD105^+^ cells by LC MS/MS, also showed divergence at the mRNA level. Specifically, Anxa1 mRNA was detected at equivalent levels in CD105^+^ and CD105^−^ cells (1.02±0.08) while Anxa4 mRNA was 3.1±0.6 fold up-regulated in CD105^+^ cells. To ensure that the detected differences were not due to errors in our quantitative proteomic method, we used Western blotting to verify the expression level of protein isoforms to which reliable antibodies were available. Densitometry analysis determined that the amount of Septin6, Septin7, Hmgb2 and Hmgb3 detected by Western blot was equivalent to that detected by LC MS/MS quantification (Fig. 3B). Overall, direct comparison of mRNA and protein quantification in MSC demonstrates the importance of utilizing both transcriptomic and proteomic analyses for investigating regulatory pathways in complex biological systems, such as stem cells.

To provide further insights into protein isoforms that may play in important role in the biology of MSC, we used IPA to analyze isoforms that were up- or down-regulated in CD105^+^ cells by at least twofold. Figure 3C depicts the only network identified from this small number of proteins with statistically significant score (>50). Within this network β- and α-tubulin isoforms seemed to play a central role. The significance of this finding with regard to MSC function requires further investigation.

## 4 Concluding remarks

Many proteins have several isoforms, each of which may have related, distinct or even opposite functions. The ability to distinguish and quantify isoforms in complex samples by LC MS/MS remains a technical challenge due to the wide dynamic range of protein concentrations, high sequence homology, and in the case of chemical labeling, the presence of peptides that do not contain the specific amino acid being labeled. We have been able to address some of these challenges through the use of reductive dimethylation labeling on intact proteins and combining data-dependent LC MS/MS scanning of the fixed charge states with Mascot-based data mining. Using this method, we were able to quantitatively compare the expression of 123 highly homologous proteins in MSC-enriched and depleted cell populations and identify 34 isoforms that were differentially expressed in stem cells. To our knowledge, this is the first study to identify quantitative differences in protein isoforms that are specific to MSC compared with stroma that lack stem cell activity. The work provides a list of candidate proteins that may be suitable markers of MSC in culture or that are important in regulating the function of these clinically important cells. In all, our study has demonstrated the feasibility of quantifying a wide range of protein isoforms in complex cell extracts using dimethylation labeling of intact proteins.

## Abbreviations

Anp32: acidic (leucine-rich) nuclear phosphoprotein 32
CCT: chaperonin-containing TCP-1
Hmg: high mobility group
hnRNP: heterogeneous nuclear ribonucleoprotein
IPA: ingenuity pathway analysis
MSC: mesenchymal stem cells
Psmd: proteasome 26S non-ATPase subunit
TIC: total ion chromatogram
TPM: tropomyosin
UBE: ubiquitin-conjugating enzyme
XIC: extracted ion chromatogram

## supplementary-material

Detailed facts of importance to specialist readers are published as ”Supporting Information”. Such documents are peer-reviewed, but not copy-edited or typeset. They are made available as submitted by the authors.
