# Supplementary material for: Quantification of protein isoforms in mesenchymal stem cells by reductive dimethylation of lysines in intact proteins
Source: Proteomics. 2012 Jan 13;12(3):369–79. doi: 10.1002/pmic.201100308 (PMC3440571; doi:10.1002/pmic.201100308)
Supplement: Supplementary file 1 [file pmic0012-0369-SD1.pdf]

Table S1. Observation of the Dimethylated Peptides from Trypsin-digested Bovine Apotransferrin

RT (min)	m/z (Obs.)	Mr(Expt)	Mr(Calc.)	ppm	Score	Position	Peptide Sequence	Intensity (TIC)
8.98	492.5686 (3+)	1474.6840	1474.6834	1	20	527-539	K.GTGKECVPNSNER.Y Dimethyl (K4)	3 %
	494.5811 (3+)	1480.7215	1480.7211	0				
	738.3500 (2+)	1474.6854	1474.6834	1	47			
	741.3688 (2+)	1480.7230	1480.7211	1	52			
9.41	628.8489 (2+)	1255.6832	1255.6819	1	25	132-142	R.GKKSCHTGLGR.S Dimethyl (K2,K3)	8%
	634.8858 (2+)	1267.7570	1267.7572	0	28			
9.48	513.5967 (3+)	1537.7683	1537.7671	1	67	595-607	R.KPVTDAENCHLAR.G Dimethyl (K1)	22%
	515.6092 (3+)	1543.8058	1543.8047	1	79			
	769.8920 (2+)	1537.7694	1737.7671	2	64			
	772.9104 (2+)	1543.8062	1543.8047	1	68			
10.33	315.6827 (2+)	629.3498	629.3497	0	47	43-47	R. ENVLR.I lysine-free peptide	2%
12.00	353.1896 (2+)	704.3646	704.3639	1	39	683-688	R. AMTNLR.Q lysine-free peptide	7%
13.86	625.3269 (2+)	1248.6392	1248.6384	1	46	659-669	R. DDTKCLASIAK.K Dimethyl (K4)	3%
	628.3457 (2+)	1254.6768	1254.6760	1	42			
14.27	826.4276 (2+)	1650.8406	1650.8399	0	65	582-594	K. KENFEVLCKDGTR.K 2 Dimethyl (K1, K9)	2%
	832.4654 (2+)	1662.9162	1662.9152	1	61			
14.80	998.4618 (2+)	1994.9090	1994.9091	0	78	27-42	R.WCTISTHEANKCASFR.E Dimethyl (K11)	24%
	1001.4810 (2+)	2000.9474	2000.9467	0	83			
	665.9775 (3+)	1994.9107	1994.9091	1	41			
	667.9898 (3+)	2000.9476	2000.9467	0	32			
15.70	468.7545 (2+)	935.4944	935.4937	1	67	608-616	R.GPNHAVVSR.K lysine-free peptide	16%
16.31	740.3535 (4+)	2957.3849	2957.3833	1	32	351-374	R.ESKPPDSSKDECMVKWCAIGHQER.T 3 Dimethyl (K3, K9, K15)	5%
	744.8819 (4+)	2975.4963	2975.4963	0				
	986.8016 (3+)	2957.3830	2957.3833	0	57			
	992.8395 (3+)	2975.4950	2975.4963	0	53			
17.40	504.2490 (3+)	1509.7252	1509.7245	0	37	244-255	R.KNYELLCGDNTR.K Dimethyl (K1)	15%
	506.2617 (3+)	1515.7633	1515.7622	1	34			
	755.8698 (2+)	1509.7250	1509.7245	0	60			
	758.8888 (2+)	1515.7630	1515.7622	1	73			
	981.9932 (2+)	1961.9718	1961.9703	1	40	689-704	R.QCSTSKLLEACTFHKP.- (protein C-terminus) 2Dimethyl (K6, K15)	
	988.0308 (2+)	1974.0470	1974.0456	1	51			
	654.9978 (3+)	1961.9716	1961.9703	1	49			
	659.0233 (3+)	1974.0481	1974.0456	1	52			

18.32	549.2564 (2+)	1096.4982	1096.4978	0	55	540-548	R.YYGYTGAFR.C lysine-free peptide	100%
19.99	649.3218 (3+)	1944.9436	1944.9438	0	57	689-704	R.QCSTSKLLEACTFHKP. - (protein C-terminus) Gln->pyro-Glu, 2Dimethyl (K6, K15)	25%
	653.3447 (3+)	1957.0123	1957.0191	3	45			
20.70	707.6260 (4+)	2826.4749	2826.4738	0	38	143-166	R.SAGWNIPMAKLYKELPDPQESIQR.A 2 Dimethyl (K10, K13)	22%
	710.6551 (4+)	2838.5913	2838.5892	1	41			
	943.1660 (3+)	2826.4763	2826.4738	1	37			
	947.2046 (3+)	2838.5920	2838.5892	1	35			
22.82	642.3160 (4+)	2565.2349	2565.2356	0	70	256-277	R.KSVDDYQECYLAMVPSHAVVAR.T Dimethyl (K1)	48%
	643.8256 (4+)	2571.2733	2571.2732	0	69			
	856.0859 (3+)	2565.2359	2565.2356	0	65			
	858.0988 (3+)	2571.2746	2571.2732	1	56			
23.52	768.0180 (3+)	2301.0322	2301.0307	1	50	494-513	K.INNCKFDEFFSAGCAPGSPR.T Dimethyl (K5)	11%
	770.0304 (3+)	2307.0694	2307.0683	0	70			
24.53	798.7391 (3+)	2393.1955	2393.1937	1	42	663-682	K.CLASIAKKTYDSYLGDDYVR.A 2 Dimethyl (K7, K8)	2%
	802.7641 (3+)	2405.2705	2405.2690	1	39			
	599.3049 (4+)	2393.1905	2393.1937	-1	36			
	602.3250 (4+)	2405.2709	2405.2690	1	44			
26.23	1016.1520 (3+)	3045.4342	3045.4324	1	90	217-243	K.CLMEGAGDVAFVKHSTVFDNLPNPEDR.K Dimethyl (K13)	10%
	1018.1650 (3+)	3051.4732	3051.4701	1	75			
	762.3657 (4+)	3045.4337	3045.4324	0	41			
	763.8757 (4+)	3051.4737	3051.4701	1				
27.24	961.1479 (3+)	2880.4219	2880.4215	0	31	659-682	R.DDTKCLASIAKKTYDSYLGDDYVR.A 3 Dimethyl (K4, K11,K12)	38%
	967.1859 (3+)	2898.5352	2898.5345	0	41			
	721.1129 (4+)	2880.4203	2880.4215	0	35			
	725.6413 (4+)	2898.5361	2898.5345	1	29			
29.62	879.1664 (4+)	3512.6365	3512.6341	1	33	630-658	K.QQDDFGKSVTDCTSNFCLFQSNSKDLLFR.D 2 Dimethyl (K7, K24)	7%
	882.1851 (4+)	3524.7113	3524.7094	1				
	889.7572 (3+)	2666.2498	2666.2469	1	57	637-658	K.SVTDCTSNFCLFQSNSKDLLFR.D Dimethyl (K17)	
	891.7692 (3+)	2672.2858	2672.2845	0				
	845.7714 (4+)	2534.2924	2534.2918	0	55	278-299	R.TVGGKEDVIWELLNHAQEHFGK.D Dimethyl (K5)	
	847.7840 (4+)	2540.3302	2540.3294	0	56			
31.00	736.7152 (3+)	2207.1238	2207.1231	0	38	480-498	R.TAGWNIPMGLLYSKINNCK.F Dimethyl (K14)	1%
	738.7283 (3+)	2213.1614	2213.1607	0				
32.20	982.7827 (3+)	2945.3263	2945.3245	1	69	380-405	R.WSGFSGGAIECETAENTEECIAKIMK.G Dimethyl (K23)	2%
	984.7953 (3+)	2951.3641	2951.3622	1				
35.07	966.2198 (4+)	3860.8501	3860.8477	1	37	480-513	R.TAGWNIPMGLLYSKINNCKFDEFFSAGCAPGSPR.N 2 Dimethyl (K14,K19)	16%
	969.2382 (4+)	3872.9237	3872.9230	0	65			

Table S2. List of High Abundance Peptides from a Tryptic Digest of Dimethylated Protein Mixture

RT (min)	m/z (Obs.)	Mr(Expt)	Mr(Calc.)	ppm	Score	Position	Peptide Sequence [*a]	Intensity (TIC)
9.48	611.3202 (2+)	1220.6258	1220.6262	0	40	25-34	R.DTH**K**SEIAHR.F Dimethyl (K4), albumin	4%
	614.3395 (2+)	1226.6644	1226.6638	1	43			
9.86	628.8489 (2+)	1255.6832	1255.6819	1	49	132-142	R.G**KK**SCHTGLGR.S 2Dimethyl (K2,K3), apotransferrin	3%
	634.8858 (2+)	1267.7570	1267.7572	0	50			
10.44	493.3114 (2+)	984.6082	984.6080	0	36	52-59	R.TQIN**K**VVR.F Dimethyl (K5), ovalbumin	10%
	496.3303 (2+)	990.6450	990.6456	-1	41			
	555.8170 (2+)	1109.6194	1109.6193	0	52	74-82	R.HAED**K**LITR.E Dimethyl (K5), alpha-1-acid glycoprotein	
	558.8361 (2+)	1115.6576	1115.6569	1	46			
10.47	513.5967 (3+)	1537.7683	1537.7671	1	95	595-607	R.**K**PVTDAENCHLAR Dimethyl (K1), apotransferrin	11%
	515.6089 (3+)	1543.8049	1543.8047	0	77			
	769.8911 (2+)	1537.7676	1737.7671	0	66			
	772.9097 (2+)	1543.8054	1543.8047	0	77			
10.82	569.3043 (2+)	1136.5940	1136.5933	1	56	220-229	R.VASMASE**K**M**K**.I 2 Dimethyl (K8, K10) ; ovalbumin [*c]	4%
	575.3421 (2+)	1148.6696	1148.6686	1	62			
10.94	641.2544 (1+)	640.2466	-	-		-	Unknown singly charged ion peak [*b]	6%
11.85	489.7374 (2+)	977.4592	-	-		-	Unknown doubly charged ion peak [*b]	8%
12.88	569.7529 (2+)	1137.4902	-	-		-	Unknown doubly charged ion peak [*b]	10%
13.75	612.3141 (2+)	1222.6136	1222.6128	1	41	223-232	R.CASIQ**K**FGER.A Dimethyl (K6), albumin	8%
	615.3330 (2+)	1228.6514	1228.6505	1	46			
13.96	539.2810 (2+)	1076.5474	1076.5470	0	41	24-32	R.CELAAAM**K**R.H Dimethyl (K8), lysozyme	5%
	542.3002 (2+)	1082.5858	1082.5847	1	33			
14.56	515.7748 (2+)	1029.5350	1029.5342	1	38	28-36	R.QSPVDIDT**K**.A Dimethyl (K9) carbonic anhydrase 2 [*c]	1%
	518.7935 (2+)	1035.5724	1035.5719	1	52			
14.94	715.9557 (3+)	2144.8453	2144.8449	0	21	106-122	R.ETYGDMADCCE**K**QEPER.N Dimethyl (K12), albumin	6%
	717.9682 (3+)	2150.8828	2150.8825	0	22			
	1073.4300 (2+)	2144.8454	2144.8449	0	66			
	1076.4490 (2+)	2150.8834	2150.8825	0	68			
16.19	480.6089 (3+)	1438.8049	1438.8045	1	81	360-371	R.RHPEYAVSVLLR.L albumin, lysine-free peptide	10%
	481.7789 (4+)	1923.0843	1923.0863	-1	31	483-498	R.LCVLHE**K**TPVSE**K**VTK.C 2 Dimethyl (K7,K13) albumin	
	484.7981 (4+)	1935.1611	1935.1616	0	36			
	642.0361 (3+)	1923.0865	1923.0863	0	40			
	646.0613 (3+)	1935.1621	1935.1616	0	48			

17.97	536.8661 (2+)	1071.7176	1071.7168	1	22	278-285	R.**K**I**K**VYLPR.M 2 Dimethyl (K1,K3), ovalbumin	5%
	542.9034 (2+)	1083.7922	1083.7921	0	27			
18.29	504.2488 (3+)	1509.7246	1509.7245	0	46	244-255	R.**K**NYELLCGDNTR.K Dimethyl (K1) apotransferrin	6%
	506.2616 (3+)	1515.7630	1515.7622	1	44			
	755.8698 (2+)	1509.7250	1509.7245	0	76			
	758.8887 (2+)	1515.7628	1515.7622	0	73			
	515.3140 (2+)	1028.6134	1028.6131	0	54	233-241	R.AL**K**AWSVAR.L Dimethyl (K3) albumin	
	518.3329 (2+)	1034.6512	1034.6507	1	49			
18.92	665.9775 (3+)	1994.9107	1994.9091	1	36	27-42	R.WCTISTHEAN**K**CASFR.E Dimethyl (K11) apotransferrin	10%
	667.9897 (3+)	2000.9473	2000.9467	0	39			
	998.4620 (2+)	1994.9094	1994.9091	0	88			
	1001.4810 (2+)	2000.9473	2000.9467	0	96			
19.69	497.2036 (2+)	992.3926	992.3923	0	25	80-86	R.WWCNDGR.T lysozyme C, lysine-free peptide	16%
20.50	499.2456 (3+)	1494.7153	1494.7137	1	20	583-594	K.ENFEVLC**K**DGTR.K Dimethyl (K8), apotransferrin	12%
	501.2577 (3+)	1500.7496	1500.7513	-1	23			
	748.3646 (2+)	1494.7146	1494.7137	1	50			
	751.3836 (2+)	1500.7526	1500.7513	1	51			
21.34	768.6573 (4+)	3070.6001	3070.5977	1	33	483-507	R.LCVLHE**K**TPVSE**K**VT**K**CCTESLVNR.R 3 Dimethyl (K7,K13,K16); albumin	8%
	773.1849 (4+)	3088.7105	3088.7107	0	39			
	1024.5410 (3+)	3070.6012	3070.5977	1	48			
	1030.5770 (3+)	3088.7092	3088.7107	0	78			
22.93	654.9975 (3+)	1961.9707	1961.9703	0	52	689-704	R.QCSTS**K**LLEACTFH**K**P.- (protein C-terminus) 2Dimethyl (K6, K15), apotransferrin	10%
	659.0228 (3+)	1974.0466	1974.0456	0	42			
	981.9924 (2+)	1961.9702	1961.9703	0	60			
	988.0299 (2+)	1974.0452	1974.0456	0	56			
23.95	458.9298 (3+)	1373.7676	1373.7667	1	34	43-54	R.LIFAG**K**QLEDGR.T Dimethyl (K6), ubiquitin	18%
	460.9425 (3+)	1379.8057	1379.8043	1	31			
	687.8908 (2+)	1373.7670	1373.7667	0	74			
	690.9097 (2+)	1379.8048	1379.8043	0	74			
	549.2564 (2+)	1096.4982	1096.4978	0	69	540-548	R.YYGYTGAFR.C apotransferrin, lysine-free peptide	
24.38	454.5714 (3+)	1360.6924	1360.6922	0	34	133-143	R.C**K**GTDVQAWIR.G Dimethyl (K2), lysozyme C	24%
	456.5839 (3+)	1366.7282	1366.7298	-1	36			
	681.3539 (2+)	1360.6932	1360.6922	1	40			
	684.3727 (2+)	1366.7308	1366.7298	1	40			
25.00	556.6615 (3+)	1666.9611	1666.9618	0	59	437-451	R.**K**VPQVSTPTLVEVSR.S Dimethyl (K2), albumin	9%
	558.6739 (3+)	1672.9983	1672.9994	-1	66			
	834.4888 (2+)	1666.9630	1666.9618	1	67			
	837.5067 (2+)	1672.9988	1672.9994	0	72			

27.00	649.3221 (3+)	1944.9445	1944.9438	0	81	689-704	R.QCSTS**K**LLEACTFH**K**P. - (protein C-terminus) Gln-	14%
	653.3460 (3+)	1957.0162	1957.0191	-1	59		>pyro-Glu, 2Dimethyl (K6, K15), apotransferrin	
	973.4809 (2+)	1944.9472	1944.9438	2	61			
	979.5176 (2+)	1957.0195	1957.0191	0	60			
28.60	723.3561 (1+)	722.3483	-	-		-	Unknown singly charge ion peak (*b)	11%
27.87	805.4330 (2+)	1608.8514	1608.8511	0	44	141-154	2 R.NDIAA**K**Y**K**ELGFQG.- (protein C-terminus) ,	16%
	811.4709 (2+)	1620.9272	1620.9264	1	45		2 Dimethyl (K6,K8), myoglobin	
29.56	877.4208 (2+)	1752.8270	1752.8278	0	106	64-79	R.NTDGSTDYGILQINSR.W lysozyme C, lysine-free	43%
	882.4281 (4+)	3525.6833	3525.6834	0	43	59-89	R.MVNNGHSFNVEYDDSQD**K**AVL**K**DGPLTGTYR.L	
	885.4469 (4+)	3537.7585	3537.7588	0	36		2 Dimethyl (K18, K22), carbonic anhydrase 2	
	1176.2349 (3+)	3525.6812	3525.6834	-1	81			
	1180.2594 (3+)	3537.7547	3537.7588	-1	78			
30.48	862.0587 (3+)	2583.1543	2583.1528	1	62	90-111	R.LVQFHFHWGSSDDQGSEHTVDR.K carbonic anhydrase	3%
							2, lysine-free peptide	
30.77	844.4231 (2+)	1686.8316	1686.8325	-1	69	128-143	R.GGLEPINFQTAADQAR.E, ovalbumin, lysine-free peptide	100%
31.76	720.0674 (3+)	2157.1804	2157.1793	0	54	55-72	R.TLSDYNIQ**K**ESTLHLVLR.L Dimethyl (K9), ubiquitin	22%
	722.0797 (3+)	2163.2173	2163.2170	0	56			
	1079.5970 (2+)	2157.1794	2157.1793	0	70			
	1082.0314 (2+)	2163.2154	2163.2170	0	45			
	642.3163 (4+)	2565.2361	2565.2356	0	52	256-277	R.**K**SVDDYQECYLAMVPSHAVVAR.T Dimethyl (K1),	
	643.8257 (4+)	2571.2737	2571.2732	0	43		apotransferrin	
	856.0858 (3+)	2565.2356	2565.2356	0	55			
	858.0989 (3+)	2571.2749	2571.2732	1	69			
34.88	566.3010 (1+)	565.2932	-	-		-	Unknown singly charged ion peaks (*b)	9%
	572.3387 (1+)	571.3309	-	-		-		
35.29	556.9789 (3+)	1667.9149	1667.9147	0	28	115-127	R.EHFVDLLLS**K**HFR.T Dimethyl (K10), alpha-1-acid	8%
	558.9916 (3+)	1673.9530	1673.9524	0	24		glycoprotein	
	834.9649 (2+)	1667.9152	1667.9147	0	61			
	837.9836 (2+)	1673.9526	1673.9524	0	67			
36.43	858.4070 (2+)	1714.7994	1714.7985	1	75	165-178	R.LSFNPTQLEEQCHI.- (protein C-terminus), beta-	70%
							lactoglobulin precursor ; lysine-free peptide	
37.38	716.4095 (1+)	715.4017	-	-		-	Unknown singly charged ion peak (*b)	16%
	1055.4623 (1+)	1054.4545	-	-		-	Unknown singly charged ion peak (*b)	
39.34	929.9872 (2+)	1857.9598	1857.9585	1	104	144-159	R.ELINSWVESQTNGIIR.N, ovalbumin, lysine-free peptide	39%
40.28	721.1129 (4+)	2880.4203	2880.4219	-1	22	659-682	R.DDT**K**CLASIA**KK**TYDSYLGDDYVR.A 3 Dimethyl	16%
	725.6411 (4+)	2898.5353	2898.5345	0	21		2H(6) (K4, K11, K12), apotransferrin	
	961.1479 (3+)	2880.4219	2880.4219	1	42			
	967.1860 (3+)	2898.5362	2898.5345	1	38			

41.25	770.4098 (3+)	2308.2059	2308.2063	0	52	86-105	R.DILNQIT**K**PNDVYSFSLASR.L Dimethyl (K8); ovalbumin	17%
	772.4218 (3+)	2314.2436	2314.2439	0	55			
	1155.1110 (2+)	2308.2074	2308.2063	1	85			
	1158.1300 (2+)	2314.2454	2314.2439	1	79			
41.89	921.7701 (3+)	2762.2885	2762.2871	1	81	40-63	R.GYSLGNWVCAA**K**FESNFNTQATNR.N Dimethyl (K12), lysozyme C	38%
	923.7826 (3+)	2768.3260	2768.3247	0	78			
	929.9871 (2+)	1857.9596	1857.9585	1	104	144-159	R.ELINSWVESQTNGIIR.N , ovalbumin, lysine-free peptide	
42.69	713.3813 (2+)	1424.7480	1424.7452	2	70	62-73	R.AIQAAFFYLEPR.H alpha-1-acid glycoprotein, lysine-free peptide	18%
43.46	930.4788 (2+)	1858.9420	1858.9425	0	73	144-159	R.ELINSWVESQTNGIIR.N Deamidated (N) , ovalbumin, lysine-free peptide with a NG motif	16%
	781.1013 (3+)	2340.2821	2340.2828	0	82	57-76	R.VYVEEL**K**PTPEGDLEILLQK.W Dimethyl (K7); beta-lactoglobulin,	
	783.1146 (3+)	2346.3220	2346.3205	1	75			
44.32	771.3978 (3+)	2311.1716	2311.1704	0	74	201-219	R.VTEQES**K**PVQMMYQIGLFR.V Dimethyl (K7) ovalbumin	5%
	773.4111 (3+)	2317.2115	2317.2081	1	52			
45.00	570.9150 (5+)	2849.5386	2849.5360	1	26	141-164	R.TPEVDDEALE**K**FD**K**AL**K**ALPMHIR.L 3 Dimethyl (K11, K14, K17), beta-lactoglobulin	24%
	574.5371 (5+)	2867.6464	2867.6490	-1	28			
	950.8517 (3+)	2849.5333	2849.5360	-1	64			
	956.8906 (3+)	2867.6500	2867.6490	0	82			
	810.1991 (4+)	3236.7673	3236.7656	1	24	28-57	R.QSPVDIDT**K**AVVQDPAL**K**PLALVYGEATSR.R 2 Dimethyl (K9, K18), carbonic anhydrase 2	
	813.2173 (4+)	3248.8401	3248.8409	0	25			
	1079.9300 (3+)	3236.7682	3236.7656	1	51			
	1083.9540 (3+)	3248.8402	3248.8409	0	41			
46.12	704.7118 (3+)	2111.1136	2111.1125	1	21	112-127	R.YPILPEYLQCV**K**ELYR.G Dimethyl (K12), ovalbumin	12%
	706.7245 (3+)	2117.1500	2117.1501	0	29			
	1056.5640 (2+)	2111.1134	2111.1125	0	58			
	1059.5820 (2+)	2117.1494	2117.1501	0	77			
	772.7966 (5+)	3858.9466	3858.9589	-3	71	135-166	R.LHSM**K**EGIHAQQ**K**EPMIGVNQELAYFYPELFR.Q 2 Dimethyl (K5, K13), alpha-S1-casein	
	775.2147 (5+)	3871.0344	3871.0341	0				
	965.7454 (4+)	3858.9503	3858.9589	-2	60			
	968.7679 (4+)	3871.0403	3871.0341	2	56			
47.25	634.3566 (2+)	1266.6986	1266.6972	1	68	106-115	R.YLGYLEQLLR.L alpha-S1-casein, lysine-free peptide	21%
48.24	862.9213 (2+)	1723.8280	1723.8273	0	115	469-482	R.MPCTEDYLSLILNR.L, albumin, lysine-free peptide	52%
	720.4102 (2+)	1438.8058	1438.8045	0	80	360-371	R.RHPEYAVSVLLR.L, albumin, lysine-free peptide	
48.80	1074.2540 (3+)	3219.7402	3219.7391	0	58	28-57	R.QSPVDIDT**K**AVVQDPAL**K**PLALVYGEATSR.R Gln->pyro-Glu (N-term Q); 2 Dimethyl (K9, K18); carbonic anhydrase 2	8%
	1078.2790 (3+)	3231.8152	3231.8144	0	51			

49.83	706.8844 (2+)	1411.7542	1411.7540	0	22	38-49	R.FFVAPFPEVFG**K**.E Dimethyl (K12); alpha-S1-casein (*c)	1%
	709.9041 (2+)	1417.7936	1417.7917	1	30			
52.95	966.2198 (4+)	3860.8501	3860.8477	1	53	480-513	R.TAGWNIPMGLLYS**K**INNC**K**FDEFFSAGCAPGSPR.N 2 Dimethyl (K14,K19), apotransferrin	14%
	969.2379 (4+)	3872.9225	3872.9230	0	50			
	1287.9619 (3+)	3860.8622	3860.8477	3	80			
	1291.9839 (3+)	3872.9282	3872.9230	1	86			
53.50	651.3502 (4+)	2601.3717	2601.3679	1	30	361-382	R.ADHPFLFCI**K**HIATNAVLFFGR.C Dimethyl (K10) , ovalbumin	40%
	652.8586 (4+)	2607.4053	2607.4055	0	32			
	868.1312 (3+)	2601.3718	2601.3679	2	40			
	870.1432 (3+)	2607.4078	2607.4055	1	50			
55.26	729.3948 (3+)	2185.1626	2185.1605	1	63	199-217	R.DMPIQAFLLYQEPVLGPVR.G , beta-casein, lysine-free peptide	2%
	1093.5880 (2+)	2185.1614	2185.1605	0	81			
60.13	1088.0404 (4+)	4348.1303	4348.1226	2		92-130	R.NLCNIPCSALLSSDITASVNCA**KK**IVSDGNGMNAWVAWR.N 2 Dimethyl (K23, K24), lysozyme C	5%
	1091.0590 (4+)	4360.2069	4360.1978	2	23			
61.75	865.7067 (4+)	3458.7955	3458.7947	0	29	2-32	M.GLSDGEWQQVLNVWG**K**VEADIAGHGQEVLIR.L , Dimethyl (K16), N-terminal dimethylation, myoglobin	39%
	868.7254 (4+)	3470.8703	3470.8699	0	39			
	1153.9386 (3+)	3458.7923	3458.7947	-1	120			
	1157.9642 (3+)	3470.8691	3470.8699	0	128			
62.40	1174.3653 (4+)	4693.4299	4693.4290	0	35	160-200	R.NVLQPSSVDSQTAMVLVNAIVF**K**GLWE**K**AF**K**DEDTQAMPFR.V 3 Dimethyl (K23, K28, K31), ovalbumin	18%
	1178.8940 (4+)	4711.5469	4711.5418	1	36			
65.38	1090.8477 (4+)	4359.3595	4359.3975	-9	31	1-40	-.LIVTQTM**K**GLDIQ**K**VAGTWYSLAMAASDISLLDAQSAPLR.V 2 Dimethyl (K8,K14); oxidation, N-terminal dimethylation, beta-lactoglobulin	7%
	1095.3750 (4+)	4377.4687	4377.5103	-9	34			
67.79	880.9455 (4+)	3519.7529	3519.7465	2	84	21-51	K.VHHANENIFYCPIAIMSALAMVYLGA**K**DSTR.T Dimethyl (K27), ovalbumin	4%
	882.4536 (4+)	3525.7853	3525.7841	0	45			
68.67	968.8172 (6+)	5806.8563	-	-		-	Too large peptide, not retrieved by database search	5%
	972.3392 (6+)	5827.9883	-	-		-		

* a) Dimethylated lysine labelled in bold red and underline (**K**).

b) Unknown peaks were not retrieved by database search because the singly charged ions were excluded by MS/MS data acquisition. Some of them might be the trypsin autolysis products or other contaminates.

c) Unexpected peptide pairs cleaved at the dimethylated lysine residue (at 10.82 min; 14.56min and 49.83min).

Table S3, Identification of Tropomysin(TPM) Peptides from an Un-modified MSC Nuclear Extract

Protein		Sequence	
TPM1	1	MDAIKKKMQMLK**LDKENALDRAEQAEADKK**AAEDR**SKQLEDELVSLQK**KLKGTEDELDKYSEALKDAQEKLELAEK**KATD**	80
TPM2	1	MDAIKKKMQMLK**LDKENAIDRAEQAEADKK**QAEDRCKQLEEEQQALQKKLKGTEDEVEKYSESVKDAQEKLEQAEK**KATD**	80
TPM3	1	MAGTTT--------IEAVKRKIQVLQQQADDAEERAER-------LQR-------EVE---------GER-RAREQ----	44
TPM4	1	MAGLNS--------LEAVKR**KIQALQQQADDAEDRAQG-------LQR**-------ELD---------GER-ERREK----	44
TPM1	81	**AEADVASLNRRIQLVEEELDRAQERLATALQKLEEAEKAADESER**GMKVIESR**AQKDEEKMEIQEIQLK**EAK**HIAEDADR**	160
TPM2	81	**AEADVASLNRRIQLVEEELDRAQERLATALQKLEEAEKAADESER**GMKVIENRAMKDEEKMELQEMQLKEAKHIAEDSDR	160
TPM3	45	AEAEVASLNRR**IQLVEEELDRAQERLATALQKLEEAEKAADESER**GMKVIENR**ALKDEEKMELQEIQLK**EAKHIAEEADR	124
TPM4	45	AEGDAAALNRR**IQLLEEELDRAQEQLATALQNLEEAEKAADESER**GMKVIENRAMKDEEKMEILEMQLKEAK**HITDEADR**	124
TPM1	161	**KYEEVAR**K**LVIIESDLERAEER**AELSEGQVR**QLEEQLR**IMDQTLKALMAAEDKYSQKEDKYEEEIKVLSDKLKEAETRAE	240
TPM2	161	**KYEEVAR**K**LVILEGELERSEER**AEVAESRAR**QLEEELR**TMDQALKSLIASEEEYSTKEDKYEEEIKLLEEKLKEAETRAE	240
TPM3	125	**KYEEVAR**K**LVIIEGDLERTEER**AELAESRCREMDEQIRLMDQNLKCLSAAEEKYSQKEDKYEEEIKILTDKLKEAETRAE	204
TPM4	125	**KYEEVAR**KLVILEGELKRAEERAEVSELK**CGDLEEELK**NVTNNLKSLEAASEKYSEKEDKYEEEIKLLSDKLKEAETRAE	204
TPM1	241	FAERSVTKLEKSIDDLEEKVAHAKEENLSMHQMLDQTLLELNNM	284
TPM2	241	FAERSVAKLEKTIDDLEETLASAKEENVEIHQTLDQTLLELNNL	284
TPM3	205	FAERSVAKLEK**TIDDLEDKLK**CTKEEHLCTQRMLDQTLLDLNEM	248
TPM4	205	FAERTVSKLEKTIDDLEEK**LAQAKEENVGLHQTLDQTLNELNCI**	248

Protein	Peptide	m/z(charge)	Mr(expt)	Mr(calc)	ppm	Score	Miss	Sequence	Unique
TPM1	13-30	511.7629(4+)	2043.0225	2043.0232	0	57	3	K.LDKENALDRAEQAEADKK.A	
	36-48	506.2786(3+)	1515.8141	1515.8144	0	24	1	R.SKQLEDELVSLQK.K	U
		758.9145(2+)	1515.8145	1515.8144	0	88	1		
	77-90	487.5834(3+)	1459.7282	1459.7267	1	30	1	K.KATDAEADVASLNR.R	
		730.8705(2+)	1459.7265	1459.7267	0	120	1		
	77-91	539.6171(3+)	1615.8279	1615.8278	0	41	2	K.KATDAEADVASLNRR.I	
	78-90	666.8231(2+)	1331.6317	1331.6317	0	64	0	K.ATDAEADVASLNR.R	
	78-91	496.9187(3+)	1487.7342	1487.7328	1	24	1	K.ATDAEADVASLNRR.I	
		744.8732(2+)	1487.7308	1487.7328	-1	56	1		
	92-101	622.3287(2+)	1242.6428	1242.6456	-2	77	0	R.IQLVEEELDR.A	
	92-105	576.6357(3+)	1726.8852	1726.8849	0	30	1	R.IQLVEEELDRAQER.L	
		864.4498(2+)	1726.8840	1726.8850	-1	48	1		

	106-118	481.9400 (3+)	1442.7983	1442.7980	0	26	1	R.LATALQKLEEAEK.A	
		722.4063(2+)	1442.7981	1442.7980	0	78	1		
	106-125	551.2871(4+)	2201.1193	2201.1175	1	45	2	R.LATALQKLEEAEKAADESER.G	
		734.7132(3+)	2201.1161	2201.1175	-1	71	2		
	134-139	490.7561(4+)	1958.9931	1958.9983	-3	15	2	R.AQKDEEKMEIQEIQLK.E	U
		654.0068(3+)	1958.9969	1958.9983	-1	56	2		
	153-160	463.7193(2+)	925.4241	925.4253	-1	28	0	K.HIAEDADR.K	U
	161-167	447.7377(3+)	893.4609	893.4606	0	28	1	R.KYEEVAR.K	
	169-182	557.9689(3+)	1670.8850	1670.8839	1	24	1	K.LVIIESDLERAEER.A	
	192-198	458.7401(2+)	915.4646	915.4661	-1	60	0	R.QLEEQLR.I	
TPM2	13-30	511.7629(4+)	2043.0225	2043.0232	0	57	3	K.LDKENALDRAEQAEADKK.A	
	77-90	487.5834(3+)	1459.7282	1459.7267	1	30	1	K.KATDAEADVASLNR.R	
		730.8705(2+)	1459.7265	1459.7267	0	120	1		
	77-91	539.6171(3+)	1615.8279	1615.8278	0	41	2	K.KATDAEADVASLNRR.I	
	78-90	666.8231(2+)	1331.6317	1331.6317	0	64	0	K.ATDAEADVASLNR.R	
	78-91	496.9187(3+)	1487.7342	1487.7328	1	24	1	K.ATDAEADVASLNRR.I	
		744.8732(2+)	1487.7308	1487.7328	-1	56	1		
	92-101	622.3287(2+)	1242.6428	1242.6456	-2	77	0	R.IQLVEEELDR.A	
	92-105	576.6357(3+)	1726.8852	1726.8849	0	30	1	R.IQLVEEELDRAQER.L	
		864.4498(2+)	1726.8840	1726.8850	-1	48	1		
	106-118	481.9400 (3+)	1442.7983	1442.7980	0	26	1	R.LATALQKLEEAEK.A	
		722.4063(2+)	1442.7981	1442.7980	0	78	1		
	106-125	551.2871(4+)	2201.1193	2201.1175	1	45	2	R.LATALQKLEEAEKAADESER.G	
		734.7132(3+)	2201.1161	2201.1175	-1	71	2		
	161-167	447.7377(3+)	893.4609	893.4606	0	28	1	R.KYEEVAR.K	
	169-182	557.9689(3+)	1670.8850	1670.8839	1	24	1	K.LVILEGELERSEER.A	
	192-198	458.7401(2+)	915.4646	915.4661	-1	60	0	R.QLEEQLR.I	
TPM3	56-65	622.3287(2+)	1242.6428	1242.6456	-2	77	0	R.IQLVEEELDR.A	
	56-69	576.6357(3+)	1726.8852	1726.8849	0	30	1	R.IQLVEEELDRAQER.L	
		864.4498(2+)	1726.8840	1726.8850	-1	48	1		
	70-82	481.9400 (3+)	1442.7983	1442.7980	0	26	1	R.LATALQKLEEAEK.A	
		722.4063(2+)	1442.7981	1442.7980	0	78	1		
	70-89	551.2871(4+)	2201.1193	2201.1175	1	45	2	R.LATALQKLEEAEKAADESER.G	
		734.7132(3+)	2201.1161	2201.1175	-1	71	2		
	98-113	649.0160(3+)	1944.0245	1944.0238	0	37	2	R.ALKDEEKMELQEIQLK.E	U

	125-131	447.7377(3+)	893.4609	893.4606	0	28	1	R.KYEEVAR.K	
	133-146	557.9689(3+)	1670.8850	1670.8839	1	24	1	K.LVIIEGDLERTEER.A	
	216-225	595.3195(2+)	1188.6244	1188.6238	1	44	1	K.TIDDLEDKLK.C	U
TPM4	13-27	576.9547(3+)	1727.8406	1727.8438	-2	62	1	R.KIQALQQQADDAEDR.A	U
	13-33	794.7419(3+)	2381.2022	2381.2047	-1	87	2	R.KIQALQQQADDAEDRAQGLQR.E	U
	14-27	800.8824(2+)	1599.7502	1599.7489	1	106	0	K.IQALQQQADDAEDR.A	U
	56-65	629.3377(2+)	1256.6609	1256.6612	0	53	0	R.IQLLEEELDR.A	U
	66 - 89	882.0948(3+)	2643.2609	2643.2624	-1	86	1	R.AQEQLATALQNLEEAEKAADESER.G	U
	117-124	478.7255(2+)	955.4354	955.4359	1	34	0	K.HITDEADR.K	U
	125-131	447.7377(3+)	893.4609	893.4606	0	28	1	R.KYEEVAR.K	
	154-162	546.7475(2+)	1091.4805	1091.4805	0	53	0	K.CGDLEEELK.N	U
	224-246	951.1470(3+)	2850.4175	2850.4182	0	71	1	K.LAQAKEENVGLHQTLDQTLNELNCI.-	U
	229-248	1170.5605(2+)	2339.1054	2339.1063	0	39	0	K.EENVGLHQTLDQTLNELNCI.-	U

Proteins were digested by trypsin, and the resulting peptides were identified by the data dependent MS/MS scan of top eight ions at an UPLC run of 4 hrs. "U" is shown unique ions specific to the protein isoform, while others are shared by the isoforms.

Table S4. MS/MS Identification of the Peptides in Two Sets of Protein Isoforms

Protein	Peptide	Scan of 2+ m/z (charge)	ppm	Score	Scan of 3+ m/z (charge)	pp m	Score	Miss	Peptide sequence
Conserved dimethylated peptide pairs of tropomyosins (TPM)									
TPM1;TPM2	106 - 125	1129.5985(2+)	1	106	753.4015(3+)	1	71	2	R.LATALQKLEEAEKAADESER.G 2 Dimethyl
TPM3;TPM4	70 - 89	1135.6357(2+)	0	94	757.4260(3+)	0	62	2	(K7, K13)
TPM2	126 - 133	487.7761(2+)	0	39	325.5198(3+)	0	38	1	R.GMKVIENR.A Dimethyl (K3)
TPM3; TPM4	90 - 97	490.7946(2+)	1	33	327.5322(3+)	-1	29	1	
TPM1; TPM2	161 - 167	461.7531(2+)	0	33	308.1708(3+)	-2	27	1	R.KYEEVAR.K Dimethyl (K1)
TPM3; TPM4	125 - 131	464.7721(2+)	0	34	310.1836(3+)	-1	29	1	
Unique dimethylated peptide pairs of tropomyosins (TPM)									
TPM1	22 - 35	794.4022(2+)	0	82	529.9372(3+)	0	35	2	R.AEQAEADKKAAEDR.S 2 Dimethyl (K8, K9)
		800.4391(2+)	-1	80	533.9625(3+)	0	34	2	
	126 - 133	474.2701(2+)	-1	29	316.5162(3+)	0	37	1	R.GMKVIESR.A Dimethyl (K3)
		477.2896(2+)	0	25	318.5285(3+)	-1	33	1	
	168 - 178	671.8997(2+)	-1	60	448.2692(3+)	-1	49	1	R.KLVIIESDLER.A Dimethyl (K1)
		674.9197(2+)	0	58	450.2819(3+)	0	45	1	
TPM2	22 - 35	822.9127(2+)	0	101	548.9445(3+)	0	36	2	R.AEQAEADKKQAEDR.C 2 Dimethyl (K8, K9)
		828.9512(2+)	1	97	552.9698(3+)	0	32	2	
	168 - 178	663.9026(2+)	-1	62	442.9382(3+)	1	28	1	R.KLVILEGELER.S Dimethyl (K1)
		666.9211(2+)	-1	52	444.9502(3+)	-1	31	1	
TPM3	13 - 27	899.9688(2+)	0	117	600.3138(3+)	-1	52	1	R.KIQVLQQQADDAEER.A Dimethyl (K1)
		902.9877(2+)	1	119	602.3276(3+)	1	87	1	
	132 - 142	656.8951(2+)	0	45	438.2652(3+)	-2	41	1	R.KLVIIEGDLER.T Dimethyl (K1)
		659.9147(2+)	1	48	440.2782(3+)	-1	45	1	
TPM4	13 - 27	878.9446(2+)	0	94	586.2992(3+)	0	70	1	R.KIQALQQQADDAEDR.A Dimethyl (K1)
		881.9642(2+)	1	101	588.3115(3+)	0	58	1	
	43 - 54	636.8301(2+)	-1	65	424.8895(3+)	0	43	1	R.EKAEGDAAALNR.R Dimethyl (K2)
		639.8492(2+)	-1	80	426.9019(3+)	-1	29	1	
	66 - 89	1336.6539(2+)	0	87	891.4391(3+)	1	72	1	R.AQEQLATALQNLEEAEKAADESER.G
		1339.6765(2+)	3	84	893.4516(3+)	1	59	1	Dimethyl (K17)
	132 - 142	677.4452(2+)	0	41	451.9652(3+)	-1	58	2	R.KLVILEGELKR.A 2 Dimethyl (K1, K10)
		683.4827(2+)	0	44	455.9905(3+)	-1	51	2	
Non-lysine containing peptides (unlabeled) of tropomyosins (TPM)									
TPM1; TPM2	91 - 101	700.3792(2+)	-2	48	467.2565(3+)	-2	27	1	R.RIQLVEEELDR.A
	92 - 101	622.3301(2+)	0	74				0	R.IQLVEEELDR.A
	92 - 105				576.6345(3+)	-2	43	1	R.IQLVEEELDRAQER.L

	192 - 198	458.2483(2+)	0	47				0	R.QLEEQLR.I
	239 - 244	361.6766(2+)	-1	33				0	R.AEFAER.S
TPM1	183 - 191	494.7551(2+)	-3	55				0	R.AELSEGQVR.Q
TPM2	183 - 189	381.1931(2+)	0	31				0	R.AEVAESR.A
TPM3	43 - 54	658.8262(2+)	1	78	439.5529(3+)	0	22	0	R.EQAEAEVASLNR.R
	55 - 65	700.3792(2+)	-2	48	467.2565(3+)	-2	27	1	R.RIQLVEEELDR.A
	56 - 65	622.3301(2+)	0	74				0	R.IQLVEEELDR.A
	56 - 69				576.6345(3+)	-2	36	1	R.IQLVEEELDRAQER.L
	147 - 153	388.2016(2+)	2	26				0	R.AELAESR.C
	154 - 162				412.8542(3+)	2	32	1	R.CREMDEQIR.L
	156 - 162	460.7106(2+)	0	23				0	R.EMDEQIR.L
	203 - 208	361.6766(2+)	-1	33				0	R.AEFAER.S
	237 - 248	718.3447(2+)	1	58				0	R.MLDQTLLDLNEM.-
TPM4	28 - 33	336.6926(2+)	-1	35				0	R.AQGLQR.E
	34 - 39	359.6716(2+)	-1	49				0	R.ELDGER.E
	34 - 41	502.2431(2+)	-1	57	335.1652(3+)	1	27	1	R.ELDGERER.R
	55 - 65	707.3882(2+)	0	56	471.9285(3+)	1	59	1	R.RIQLLEEELDR.A
	56 - 65	629.3376(2+)	0	74				0	R.IQLLEEELDR.A
	203 - 208	361.6766(2+)	-1	38				0	R.AEFAER.S
Conserved dimethylated peptide pairs of 14-3-3 proteins									
14-3-3	44 – 57	766.4512(2+)	1	73	511.3035(3+)	0	47	1	R.NLLSVAY**K**NVVGAR.R Dimethyl (K8)
beta,gamma,	43 – 56	769.4697(2+)	-1	51	513.3155(3+)	-1	36	1	
Zeta	42 - 55								
Eta	43 - 56								
Unique dimethylated peptide pairs of 14-3-3 proteins									
14-3-3 beta	21 - 43				875.0636(3+)	0	85	1	R.YDDMAAAM**K**AVTEQGHELSNEER.N
					877.0770(3+)	1	78	1	Dimethyl (K9)
	63 - 73	659.3746(2+)	4	58	439.9172(3+)	0	27	1	R.VISSIEQ**K**TER.D Dimethyl (K8)
		662.3911(2+)	0	61	441.9304(3+)	1	33	1	
14-3-3	5 - 12	500.3011(2+)	0	35	333.8698(3+)	0	26	1	R.EQLVQ**K**AR.L Dimethyl (K6)
gamma		503.3191(2+)	-1	34	335.8822(3+)	0	22	1	
	20 - 42				890.0796(3+)	0	79	1	R.YDDMAAAM**K**NVTELNEPLSNEER.N
					892.0924(3+)	1	66	1	Dimethyl (K9)
	62 - 83				849.4678(3+)	1	64	3	R.VISSIEQ**K**TSADGNE**KK**IEMVR.A 3Dimethyl
					855.5040(3+)	0	63	3	(K8, K16, K17)
	133 - 143	632.8486(2+)	0	62	422.2348(3+)	0	45	1	R.YLAEVATGE**K**R.A Dimethyl (K10)
		635.8672(2+)	0	49	424.2472(3+)	0	37	1	
14-3-3 epsilon	5 - 19	895.9684(2+)	1	74	597.6473(3+)	-1	53	1	R.EDLVYQA**K**LAEQAER.Y Dimethyl (K10)

		898.9866(2+)	0	78	599.6608(3+)	1	56	1	
	43 - 56	773.4601(2+)	1	89	515.9752(3+)	0	48	1	R.NLLSVAYKNVIGAR.R Dimethyl (K8)
		776.4767(2+)	-2	75	517.9872(3+)	-1	45	1	
	62 - 83				886.5018(3+)	0	59	4	R.IISSIEQKEENKGGEDKLKMIR.E 4Dimethyl
					894.5520(3+)	0	48	4	(K8, K12, K17, K19)
	194 - 225				1209.6350(3+)	1	133	2	R.LAKAAFDDAIAELDTLSEESYKDSTLIMQLL
					1213.6593(3+)	0	141	2	R.D 2 Dimethyl (K3, K22)
	226 - 255				1160.5058(3+)	0	88	1	R.DNLTLWTSDMQGDGEEQNKEALQDVEDEN
					1162.5183(3+)	0	97	1	Q.- Dimethyl (K19)
14-3-3 zeta	19 - 41				888.0529(3+)	0	82	1	R.YDDMAACMKSVTEQGAELSNEER.N
					890.0659(3+)	1	74	1	Dimethyl (K9)
	61 - 80				777.7621(3+)	1	64	3	R.VVSSIEQKTEGAEKKQQMAR.E 3 Dimethyl
					783.7990(3+)	0	55	3	(K8, K14, K15)
	84 - 91	523.2982(2+)	0	32	349.2012(3+)	0	28	1	R.EKIETELR.D Dimethyl (K2)
		526.3167(2+)	0	36	351.2138(3+)	0	32	1	
14-3-3 eta	20 - 42				876.4047(3+)	1	57	1	R.YDDMASAMKAVTELNEPLSNEDR.N
					878.4164(3+)	0	76	1	Dimethyl (K9)
14-3-3 theta	19 - 41				892.7253(3+)	0	67	1	R.YDDMATCMKAVTEQGAELSNEER.N
					894.7369(3+)	0	71	1	Dimethyl (K9)
	42 - 55	759.4462(2+)	3	68	506.6305(3+)	-2	51	1	R.NLLSVAYKNVVGGR.R Dimethyl (K8)
		762.4612(2+)	-2	64	508.6439(3+)	0	43	1	
	84 - 91	509.2816(2+)	-2	31	339.8572(3+)	0	24	1	R.EKVESELR.S Dimethyl (K2)
		512.2996(2+)	-3	27	341.8703(3+)	0	28	1	
Non-lysine containing peptides (unlabeled) of 14-3-3 proteins									
14-3-3 beta	58 - 62	346.1851(2+)	-1	26				1	R.RSSWR.V
14-3-3 gamma	13 - 19	408.7141(2+)	0	52				0	R.LAEQAER.Y
	57 - 61	346.1851(2+)	-1	26				1	R.RSSWR.V
	228 - 247	1097.4430(2+)	0	80				0	R.DNLTLWTSDQQDDDGGEGNN.-
14-3-3 epsilon	131 - 141	628.7986(2+)	-1	51				0	R.YLAEFATGNDR.K
14-3-3 zeta	12 - 18	408.7141(2+)	0	52				0	R.LAEQAER.Y
	56 - 60	346.1851(2+)	-1	26				1	R.RSSWR.V
	223-245	1205.0008(2+)	-1	52				0	R.DNLTLWTSDTQGDEAEAGEGGEN.-
14-3-3 eta	5 - 10	393.7266(2+)	-1	29				0	R.EQLLQR.A
	13 - 19	408.7141(2+)	0	52				0	R.LAEQAER.Y
	57 - 61	346.1851(2+)	-1	26				1	R.RSSWR.V
14-3-3 theta	12 - 18	408.7141(2+)	0	52				0	R.LAEQAER.Y
	128 - 138	634.7827(2+)	0	70				0	R.YLAEVACGDDR.K

The accession numbers of proteins in the NCBI database: TPM1, gi|78000192|; TPM2, gi|66730475|; TPM3, gi|149751320|; TPM4, gi|47894398|; 14-3-3 beta, gi|3065925|; 14-3-3 gamma, gi|3065929|; 14-3-3 epsilon, gi|5803225|; 14-3-3 zeta, gi|1526539|; 14-3-3 eta, gi|6756037|; 14-3-3 theta, gi|6756039|.

Table S5. MS/MS Identification of the 4+ Charged Peptides in Two Sets of Protein Isoforms

Protein	Peptide	Scan of 4+ m/z (Obs.)	Mr(Expt)	Mr(Calc)	ppm	Score	Miss	Sequence
TPM1	91-105	471.7540(4+)	1882.9847	1882.9860	-1	33	2	R.RIQLVEEELDRAQER.L, lysine-free
	106-125	565.3027(4+)	2257.1795	2257.1802	0	65	2	R.LATALQKLEEAEKAADESER.G
		568.3215(4+)	2269.2547	2269.2555	0	72	2	2Dimethyl (K7, K13)
	134-160	827.6861(4+)	3306.7131	3306.7130	0	60	4	R.AQKDEEKMEIQEIQLKEAKHIAEDADR.K
		833.7235(4+)	3330.8627	3330.8635	0	57	4	4Dimethyl (K3, K7,K16,K19)
	168-191	700.1324(4+)	2796.4983	2796.4981	0	38	3	R.KLVIIESDLERAEERAELSEGQVR.Q
		701.6418(4+)	2802.5359	2802.5358	0	34	3	Dimethyl (K1)
TPM2	21-35	411.9604(4+)	1643.8126	1643.8114	1	29	2	R.AEQAEADKKQAEDR.C
		414.9790(4+)	1655.8847	1655.8867	-1	25	2	2Dimethyl (K8, K9)
	91-105	471.7540(4+)	1882.9847	1882.9860	-1	33	2	R.RIQLVEEELDRAQER.L, lysine-free
	106-125	565.3027(4+)	2257.1795	2257.1802	0	65	2	R.LATALQKLEEAEKAADESER.G
		568.3215(4+)	2269.2547	2269.2555	0	72	2	2Dimethyl (K7, K13)
	134-160	836.9194(4+)	3343.6463	3343.6463	0	58	4	R.AMKDEEKMELQEMQLKEAKHIAEDSDR.K
		843.2075(4+)	3367.7987	3367.7969	1	44	4	4Dimethyl (K3, K7,K16,K19)
	106-133	804.1848(4+)	3212.7079	3212.7075	0	51	4	R.LATALQKLEEAEKAADESERGMKVIENR.A
		808.7131(4+)	3230.8211	3230.8205	0	53	4	3Dimethyl (K7,K13, K23)
	245-284	1148.6117(4+)	4590.4155	4590.4168	0	39	3	R.SVAKLEKTIDDLEETLASAKEENVEIHQTLDQTLL
		1153.1405(4+)	4608.5307	4608.5298	0	36	3	ELNNL.- 3 Dimethyl (K4, K7, K20)
TPM3	13-30	539.5335(4+)	2154.1027	2154.1029	0	51	2	R.KIQVLQQQADDAEERAER.L
		541.0429(4+)	2160.1403	2160.1406	0	28	2	Dimethyl (K1)
	55-69	471.7540(4+)	1882.9847	1882.9860	-1	33	2	R.RIQLVEEELDRAQER.L , lysine-free
	70-89	565.3027(4+)	2257.1795	2257.1802	0	65	2	R.LATALQKLEEAEKAADESER.G
		568.3215(4+)	2269.2547	2269.2555	0	72	2	2Dimethyl (K7, K13)
	70-97	804.1848(4+)	3212.7079	3212.7075	0	51	4	R.LATALQKLEEAEKAADESERGMKVIENR.A
		808.7131(4+)	3230.8211	3230.8205	0	53	4	3Dimethyl (K7,K13, K23)
	98-124	827.4469(4+)	3305.7563	3305.7542	1	62	4	R.ALKDEEKMELQEIQLKEAKHIAEEADR.K ;
		833.4835(4+)	3329.9027	3329.9048	-1	55	4	4Dimethyl (K3, K7,K16,K19)
TPM4	13-33	603.3168(4+)	2409.2359	2409.2360	0	64	2	R.KIQALQQQADDAEDRAQGLQR.E
		604.8263 (4+)	2415.2739	2415.3237	0	51	2	Dimethyl (K1)
	34-54	604.0601(4+)	2412.2091	2412.2105	-1	28	4	R.ELDGERERREKAEGDAAALNR.R
		605.5698(4+)	2418.2479	2418.2482	0	21	4	Dimethyl (K11)
	43-55	357.9444(4+)	1427.7463	1427.7481	-1	29	2	R.EKAEGDAAALNRR.I

		359.4542 (4+)	1433.7876	1433.7857	1	30	2	Dimethyl (K2)
	66-89	668.8315(4+)	2671.2947	2671.2937	0	75	1	R.AQEQLATALQNLEEAEKAADESER.G
		670.3406(4+)	2677.3311	2677.3313	0	69	1	Dimethyl (K17)
	98-124	836.6796(4+)	3342.6871	3342.6874	0	42	4	R.AMKDEEKMEILEMQLKEAKHITDEADR.K
		842.7168(4+)	3366.8359	3366.8379	-1	46	4	4Dimethyl (K3, K7,K16,K19)
	132-142	339.2267(4+)	1352.8775	1352.8755	2	30	2	R.KLVILEGELKR.A
		342.2451(4+)	1364.9511	1364.9508	0	42	2	2 Dimethyl (K1, K10)
	209-248	1173.8730(4+)	4691.4607	4691.4580	0	34	4	R.TVSKLEKTIDDLEEKLAQAKEENVGLHQTLDQTL
		1179.9120(4+)	4715.6167	4715.6086	1	38	4	NELNCI.- 4 Dimethyl (K4, K7, K15, K20)
14-3-3	21-43	656.5502(4+)	2622.1695	2622.1690	0	32	1	R.YDDMAAAMKAVTEQGHELSNEER.N
beta		658.0601(4+)	2628.2091	2628.2066	1	35	1	Dimethyl (K9)
14-3-3	20-42	667.8126(4+)	2667.2191	2667.2156	1	53	1	R.YDDMAAAMKNVTELNEPLSNEER.N
gamma		669.3217(4+)	2673.2555	2673.2532	1	67	1	Dimethyl (K9)
	62-83	637.3524(4+)	2545.3783	2545.3786	0	25	3	R.VISSIEQKTSADGNEKKIEMVR.A
		641.8803(4+)	2563.4899	2563.4916	1	35	3	3 Dimethyl (K8, K16, K17)
	144-172	837.6726(4+)	3346.6591	3346.6616	1	51	2	R.ATVVESSEKAYSEAHEISKEHMQPTHPIR.L
		840.6910(4+)	3358.7327	3358.7369	-1	57	2	2Dimethyl (K9, K19)
14-3-3	20-42	686.8361(4+)	2743.3131	2743.3119	0	36	2	R.YDEMVESMKKVAGMDVELTVEER.N
epsilon		689.8549(4+)	2755.3904	2755.3871	1	24	2	2Dimethyl(K9, K10)
	62-83	665.1290(4+)	2656.4847	2656.4834	1	30	4	R.IISSIEQKEENKGGEDKLKMIR.E
		671.1666(4+)	2680.6351	2680.6340	0	22	4	4Dimethyl (K8, K12, K17, K19)
	142-170	795.6746(4+)	3178.6671	3178.6696	-1	64	2	R.KEAAENSLVAYKAASDIAMTELPPTHPIR.L
		798.6937(4+)	3190.7435	3190.7449	0	68	2	2Dimethyl (K1, K12)
	194-225	907.4786(4+)	3625.8831	3625.8801	1	61	2	R.LAKAAFDDAIAELDTLSEESYKDSTLIMQLLR.D
		910.4963(4+)	3637.9539	3637.9554	0	71	2	2 Dimethyl (K3, K22)
14-3-3	19-41	666.2916(4+)	2661.1351	2661.1356	0	91	1	R.YDDMAACMKSVTEQGAELSNEER.N
zeta		667.8020(4+)	2667.1767	2667.1733	1	85	1	Dimethyl (K9)
	61-80	583.5735(4+)	2330.2627	2330.2628	0	74	3	R.VVSSIEQKTEGAEKKQQMAR.E
		588.1014(4+)	2348.3743	2348.3758	-1	68	3	3Dimethyl (K8, K14, K15)
	128-167	1158.6002(4+)	4630.3695	4630.3742	-1	75	4	R.YLAEVAAGDDKKGIVDQSQQAYQEAFEISKKEM
		1164.6389(4+)	4654.5243	4654.5248	0	69	4	QPTHPIR.L 4Dimethyl (K11, K12, K30, K31)
14-3-3	20-42	657.5547(4+)	2626.1875	2626.1891	-1	42	1	R.YDDMASAMKAVTELNEPLSNEDR.N
eta		659.0630(4+)	2632.2207	2632.2268	2	32	1	Dimethyl (K9)
14-3-3	19-41	669.7946(4+)	2675.1471	2675.1512	-2	67	1	R.YDDMATCMKAVTEQGAELSNEER.N
theta		671.3025(4+)	2681.1787	2681.1889	4	64	1	Dimethyl(K9)

Table S6. MS/MS Measurements of Peptide ions by Scanning Top Eight Ions and Fixed-charge Ions

Protein	Number of peptide ions top eight ion scans [a]				Number of peptide ions fixed-charge ion scans [b]				Overlapped peptide ions [c]	Increased Peptide ions
	2+	3+	4+	subtotal	2+	3+	4+	subtotal	Percentage	Percentage
nuclear fraction										
TPM1	10	5	3	18	15	12	6	33	55%	183%
TPM2	9	7	0	16	15	11	2	28	57%	175%
TPM3	11	8	1	20	16	14	4	34	59%	170%
TPM4	7	7	2	16	16	10	4	30	53%	188%
14-3-3 beta	2	3	1	6	5	4	2	11	55%	183%
14-3-3 gamma	4	8	1	13	8	10	4	22	59%	169%
14-3-3 epsilon	3	4	3	10	5	4	4	13	77%	130%
14-3-3 zeta	3	5	3	11	5	6	6	17	65%	155%
14-3-3 eta	3	3	0	6	4	4	0	8	75%	133%
14-3-3 theta	2	5	0	7	4	4	0	8	87%	114%
cytosolic fraction										
TPM1	11	8	3	22	15	12	8	35	63%	159%
TPM2	11	10	0	21	15	12	12	39	54%	186%
TPM3	16	9	2	27	17	14	10	41	66%	152%
TPM4	12	11	5	28	20	16	14	50	56%	179%
14-3-3 beta	3	4	1	8	5	6	2	13	62%	163%
14-3-3 gamma	6	8	1	15	8	10	6	24	63%	160%
14-3-3 epsilon	3	4	2	9	5	10	8	23	39%	256%
14-3-3 zeta	5	6	3	14	7	8	6	21	67%	150%
14-3-3 eta	3	3	1	7	5	4	2	11	64%	157%
14-3-3 theta	2	3	0	5	6	6	2	14	36%	280%

a. Data dependent MS/MS scan of top 8 ions was performed on the nuclear and cytosolic fractions, and the peptide ions were identified by Mascot database search. No redundant ions were counted.

b. Fixed-charged ion scans were conducted by the designed charge state of 2+, 3+, or 4+ of peptides on the nuclear and cytosolic fractions, and all identified peptide ions were collected in each case. As well, no redundant ions were counted.

c. Percentage overlap refers to the ratio of total peptide ion numbers of top 8 MS/MS scans to those of the fixed-charge MS/MS scans.

Table S7. Quantitative Details of Mouse Protein Isoforms and the Closely Related Species

Accession number	Protein name	Mass (kDa)	Examples of unique dimethyl peptides	H/L (nuclear)	H/L (cytosol)
gi|78000192	TPM1, alpha	32.8	R.AEQA-EADKKAAEDR.S; R.KLVIIESDLER.A	0.98±0.07	0.95±0.03
gi|66730475	TPM2, beta	32.9	R.AEQA-EADKKQAEDR.C; R.KLVILEGELER.S	0.72±0.03	0.71±0.04
gi|149751320	TPM3, gamma	29.0	R.KIQVLQQQADDAEER.A; R.KLVIIEGDLER.T	0.72±0.06	0.70±0.07
gi|47894398	TPM4	28.5	R.KIQALQQQADDAEDR.A; R.KLVILEGELKR.A	1.11±0.09	1.16±0.06
gi|3065925	14-3-3, beta	28.1	R.YDDMAAAMKAVTEQGHELSNEERNLLSVAYKNVVGAR.R	0.56±0.04	0.58±0.05
gi|3065929	14-3-3, gamma	28.4	R.YDDMAAAMKNVTELNEPLSNEERNLLSVAYKNVVGAR.R	1.01±0.16	0.90±0.11
gi|5803225	14-3-3, epsilon	29.1	R.YDEMVESMKKVAGMDVELTVEERNLLSVAYKNVIGAR.R	1.09±0.05	1.05±0.05
gi|1526539	14-3-3, zeta	27.8	R.YDDMAACMKSVTEQGAELSNEERNLLSVAYKNVVGAR.R	0.80±0.08	0.62±0.06
gi|1526541	14-3-3, eta	28.2	R.YDDMASAMKAVTELNEPLSNEDRNLLSVAYKNVVGAR.R	1.21±0.12	1.08±0.09
gi|6756039	14-3-3, theta	27.8	R.YDDMATCMKAVTEQGAELSNEERNLLSVAYKNVVGGR.R	1.01±0.03	0.97±0.06
gi|387090	actin, alpha	41.8	R.VAPEEHPTLLTEAPLNPKANR.E	1.21±0.12	0.90±0.13
gi|187951999	actin, beta	41.7	R.VAPEEHPVLLTEAPLNPKANR.E	1.03±0.07	0.93±0.08
gi|809561	actin, gamma	41.2	R.VAPEEHPVLLTEAPLNPKANR.E	1.03±0.07	0.93±0.08
gi|61097906	actinin, alpha 1	103.1	R.VPENTMHAMQQKLEDFR.D; R.DAKGISQEQMNEFR.A	1.07±0.05	1.32±0.20
gi|11230802	actinin, alpha 4	105.0	R.VPQKTIQEMQQKLEDFR.D; R.DAKGISQEQMQEFR.A	1.10±0.02	1.12±0.15
gi|435585	myosin, Lc3	15.8	K.NKAQGTYEDYVEGLR.V; R.VFDKEGNGTVMGAEIR.H	0.59±0.06	1.33±0.14
gi|31981605	myosin, Lc4	21.3	R.NKEQGTYEDFVEGLR.V; R.VFDKESNGTVMGAELR.H	0.59±0.05	1.43±0.16
gi|33620739	myosin, Lc6	17.0	K.NKDQGTYEDYVEGLR.V; R.VFDKEGNGTVMGAEIR.H	0.59±0.05	1.32±0.06
gi|71037403	myosin, Lc, B	20.3	R.EAPIDKKGNFNYIEFTR.I	0.42±0.06	-
gi|9886714	myosin, Hc IIX	54.6	K.QIQKLEAR.V	1.10±0.09	-
gi|17978023	myosin, Hc II-A	227.4	R.ALEEAMEQKAELER.L	0.92±0.04	-
gi|6755714	transgelin 1	22.6	R.DFTDSQLQEGKHVIGLQMGSNR.G	2.08±0.06	1.50±0.16
gi|30519911	transgelin 2	22.6	R.NFSDNQLQEGKNVIGLQMGTNR.G	1.94±0.05	1.61±0.05

gi|122939192	clathrin, Lca	24.0	R.EEQTERLEALDANSR.; R.SVLISLKQAPLVH.-	1.06±0.02	0.97±0.07
gi|30794164	clathrin, Lcb	23.7	R.EEQKKRLQELDAASKVTEQEWR.E;R.SVLMSLKQTPLSR.-	0.65±0.04	0.71±0.05
gi|51491845	clathrin, Hc	193.2	R.AHIAQLCEKAGLLQR.A; R.NLQNLLILTAIKADR.T	-	0.95±0.06
gi|6754976	peroxiredoxin 1	22.9	R.GLFIIDDKGILR.Q	0.92±0.08	1.04±0.11
gi|148747558	peroxiredoxin 2	21.9	R.GLFIIDAKGVLR.Q	0.96±0.04	0.98±0.03
gi|7948999	peroxiredoxin 4	31.3	R.GLFIIDDKGVLR.Q	1.06±0.05	-
gi|6644338	peroxiredoxin 5	22.7	R.LLADPTGAFGKATDLLLDDSLVSLFGNR.R	1.18±0.08	1.20±0.10
gi|6671549	peroxiredoxin 6	25.4	R.DLAILLGMLDPVEKDANNMPVTAR.V	1.12±0.02	1.01±0.05
gi|85060507	hnRNP, A1	34.8	R.DYFEQYGKIEVIEIMTDR.G	2.00±0.16	-
gi|6754222	hnRNP, A/B	30.9	R.EYFGQFGEIEAIELPIDPKLNKR.R	1.67±0.12	-
gi|3329498	hnRNP, A2/B1	36.0	R.DYFEEYGKIDTIEIITDR.Q	0.95±0.06	-
gi|116256514	hnRNP, D	33.8	R.EYFGGFGEVESIELPMDNKTNKR.R	1.11±0.10	-
gi|19527048	hnRNP, F *	46.5	R.YIEVFKSSQEEVR.S	0.97±0.17	1.02±0.12
gi|10946928	hnRNP, H *	49.9	R.YIEIFKSSR.A	1.70±0.04	-
gi|13384620	hnRNP, K *	51.8	R.VVLIGGKPDR.V	1.89±0.02	6.11±0.21
gi|3329496	hnRNP, U *	90.5	R.GYFEYIEENKYSR.A	1.02±0.07	4.72±0.55
gi|6754208	Hmg B1	26.3	M.GKGDPKKPRGKMSSYAFFVQTCR.E	1.60±0.08	-
gi|6680229	Hmg B2	25.4	M.GKGDPNKPRGKMSSYAFFVQTCR.E	2.09±0.04	-
gi|6680231	Hmg B3	24.3	M.AKGDPKKPKGKMSAYAFFVQTCR.E	2.14±0.09	-
gi|53733821	tubulin, alpha 1A	51.3	R.TIQFVDWCPTGFKVGINYQPPTVVPGGDLAKVQR.A	-	0.51±0.04
gi|34740335	tubulin, alpha 1B	50.8	R.SIQFVDWCPTGFKVGINYQPPTVVPGGDLAKVQR.A	-	0.51±0.06
gi|91855	tubulin, beta 1	51.2	R.ISVYYNEAYGKKYVPR.A	-	0.63±0.07
gi|4507729	tubulin, beta 2	50.3	R.INVYYNEAAGNKYVPR.A	-	0.25±0.05
gi|13542680	tubulin, beta 2C	50.2	R.INVYYNEATGGKYVPR.A	-	0.57±0.14
gi|12963615	tubulin, beta 3	50.8	R.ISVYYNEASSHKYVPR.A	-	0.64±0.07
gi|7106439	tubulin, beta 5	50.1	R.ISVYYNEATGGKYVPR.A	-	0.70±0.01
gi|27754056	tubulin, beta 6	50.5	R.ISVYYNESSSKKYVPR.A	-	0.86±0.09
gi|124517663	annexin A1	39.0	R.KALLALAKGDR.C	-	0.39±0.03
gi|6996913	annexin A2	38.9	R.KLMVALAKGR.R	1.66±0.15	0.65±0.01
gi|148688409	annexin A3	36.6	R.KALLTLADGR.R	-	1.00±0.02
gi|161016799	annexin A4	36.8	R.SKPSYFAER.L; R.KVLLVLCGGDD.-	-	0.36±0.01
gi|6753060	annexin A5	35.8	R.QEIAQEFKTLFGR.D	-	0.93±0.06

gi|31981302	annexin A6	77.8	R.SKQHLR.L	-	1.68±0.05
gi|14290464	annexin A7	50.6	R.KLLLAIVGQ.-	-	1.30±0.09
gi|5902076	Sfrs 1	28.1	R.DIDLKNR.R	1.28±0.03	1.36±0.20
gi|6755478	Sfrs 2	25.8	R.RVFEKYGR.V	1.02±0.11	0.90±0.10
gi|4506901	Sfrs 3	19.7	R.VELSNGEKR.S	0.88±0.05	-
gi|122890231	Sfrs 4	58.1	R.SVSKER.E	0.85±0.14	-
gi|119226243	Sfrs 5	31.5	R.IRDIDLKR.G	0.99±0.12	-
gi|15080592	Sfrs 6	39.8	R.SPKENGKGDIKSKSR.S	1.27±0.18	-
gi|22122585	Sfrs 7	27.8	R.GLDGKVICGSR.V	1.88±0.04	-
gi|56269656	Sfrs10	27.0	R.EVFSKYGPIADVSIVYDQQSR.R	1.01±0.15	-
gi|23956214	Sfpg	76.4	R.LFVGNLPADITEDEFKR.L	2.10±0.16	-
gi|21450341	Nedd8-activating E1	61.6	R.LDKPFPELR.E	-	1.08±0.12
gi|6678483	UBA1	120.4	R.SPPSVKQNSLDEDLIR.K	-	1.90±0.19
gi|7709986	UBA2	72.5	R.TIFLNKQPNPR.K	-	1.20±0.09
gi|13384768	Ufm1	20.0	R.LKEEYQSLIR.Y	-	1.49±0.04
gi|4507773	UBE2D 1	17.1	R.IQKELSDLQR.D	-	4.35±0.16
gi|4507777	UBE2D 3	17.1	R.INKELSDLAR.D	1.81±0.14	2.03±0.11
gi|4507789	UBE2L 3	18.0	R.ADLAEEYSKDR.K	1.10±0.07	1.11±0.13
gi|4507793	UBE2N	17.4	R.LLAEPVPGIKAEPDESNAR.Y	1.32±0.08	1.37±0.21
gi|33391146	UBR2	205.1	K.KNLMTVIIK.A	-	0.90±0.13
gi|1374782	Nedd4	111.8	R.ADLLKAR.L	-	0.96±0.04
gi|51895883	Nedd4l	103.2	R.ELALVQTKTIK.K	-	0.75±0.03
gi|33563282	Psma1	30.1	R.LVSLIGSKTQIPTQR.Y	1.09±0.08	0.95±0.12
gi|8394063	Psma2	26.4	R.KLAQQYYLVYQEPIPTAQLVQR.V	1.01±0.09	1.14±0.05
gi|148704618	Psma3	26.1	R.HEIVPKDIREEAEKYAKESLKEEDESDDDNM.-	0.86±0.04	0.83±0.07
gi|6755196	Psma4	30.4	R.EKKEKEQR.E	-	1.11±0.10
gi|6755198	Psma6	27.8	R.LYQVEYAFKAINQGGLTSVAVR.G	-	0.97±0.05
gi|148675357	Psma7	25.7	R.YIASLKQRRLYQTDPSGTYHAWKANAIGR.G	-	0.91±0.06
gi|7242197	Psmb1	26.9	R.ICIVTKEGIR.E	0.95±0.07	0.87±0.05
gi|227116345	Psmb2	23.4	R.VIDKDGIHNLENIAFPKRDS.-	0.99±0.06	-
gi|6755202	Psmb3	23.6	R.LNLYELKEGRQIKPYTLMSMVANLLYEKR.F	1.10±0.04	0.91±0.08
gi|14198355	Psmb4	29.4	R.EVLEKQPVLSQTEAR.E	0.91±0.09	1.13±0.03
gi|238231384	Psmb6	25.7	R.QVLLGDQIPKFTIATLPPP.-	-	0.99±0.07

gi|6679501	Psmc1	50.6	R.LKLLKLER.I	-	1.76±0.16
gi|33859604	Psmc2	54.3	R.KIEFSLPDLEGR.T	-	1.23±0.12
gi|123226008	Psmc3	45.7	R.KMNVSPDVNYEELAR.C	-	1.53±0.07
gi|25742677	Psmc4	48.3	R.VVGSEFVQKYLGEGPR.M	-	1.24±0.05
gi|7110703	Psmc5	46.7	R.VSGSELVQKFIGEGAR.M	-	1.83±0.08
gi|74315975	Psmd1	108.7	R.LEGIVNKMFQR.C	-	2.80±0.04
gi|19882201	Psmd2	102.4	R.KNPNYDL.-	-	2.05±0.08
gi|19705424	Psmd3	61.7	R.IKAIQLEYSEAR.R; R.VYEFLDKLDVVR.S;	-	2.00±0.11
gi|148676680	Psmd5	37.4	R.VGYQAKNASTELKIR.C	-	1.47±0.12
gi|46049022	Psmd6	46.5	R.VQKLSR.V	-	0.93±0.06
gi|6754724	Psmd7	37.6	R.DIKDTTVGTLSQR.I	-	2.21±0.10
gi|31980811	Psmd10	25.8	R.ILADKSLATR.T	-	0.97±0.02
gi|72679790	Psmd11	48.2	R.LVSLYFDTKR.Y	-	2.14±0.07
gi|13385384	Psmd12	54.4	R.LQEVIETLLSLEKQTR.T	-	1.58±0.01
gi|6755210	Psmd13	43.8	R.FLGCVDIKDLPVSEQQER.A	-	1.95±0.02
gi|468546	CCT 2, beta	58.8	R.VDNIIKAAPR.K	1.31±0.05	1.84±0.08
gi|6753320	CCT3, gamma	62.1	R.GVMINKDVTHPR.M	-	3.50±0.24
gi|6753322	CCT4, delta	59.6	R.HAQGEKTTGINVR.K	1.10±0.01	1.88±0.05
gi|148666717	CCT7, eta	63.1	R.QLCDNAGFDATNILNKLR.A	2.05±0.18	1.64±0.11
gi|5295992	CCT8, theta	61.2	R.NIQACKELAQTTR.T	1.60±0.10	1.91±0.16
gi|6754816	septin 2 (Nedd5)	42.5	R.DCFKTIISYIDEQFER.Y	3.62±0.04	-
gi|5689158	septin 6	49.2	R.NEFLGELQKKEEEMR.Q	2.28±0.10	-
gi|148693353	septin 7 (CDC10)	44.9	R.QFEEEKANWEAQQR.I	2.66±0.08	-
gi|122889413	septin 9	65.2	R.RTEITIVKPQESVLR.R	1.97±0.09	-
gi|31542366	CDC2	34.8	R.EISLLKELR.H	-	1.92±0.06
gi|7949018	CDC37	46.2	R.KLKELEVAESDGQVELER.L	-	1.70±0.05
gi|149252935	TF EF1	37.1	R.NDTKEDVFVHQTAIKKNNPR.K	1.57±0.07	1.45±0.12
gi|201937	TF S-II	31.1	R.KNVLCGNIPPDLFAR.M	1.17±0.03	-
gi|28174932	Btf3l4	20.1	R.KLAEQFPR.Q	0.92±0.10	1.11±0.14
gi|226051832	Max	18.5	R.AQILDKATEYIQYMR.R	1.29±0.09	-
gi|60650308	LRR 27	61.3	K.KLQEELR.K	0.71±0.08	0.52±0.05

gi|27229082	LZIC	22.1	R.KVEILTALR.K	-	0.62±0.12
gi|13385296	Bzw1	49.4	R.KRDEKER.F	-	3.01±0.36
gi|18700032	Anp32b	31.6	R.LDCLKSLDLFGCEVTNR.S	-	1.47±0.15
gi|254587996	Anp32e	30.3	R.LPSLNKLR.K	-	7.64±0.00

14-3-3: tyrosine 3-monooxygenase/tryptophan 5-monooxygenase activation protein; hnRNP: heterogeneous nuclear ribonucleoprotein; TPM: tropomyosin; Hmg: high mobility group; Sfrs: splicing factor, arginine/serine-rich; Sfpg: splicing factor proline/glutamine rich; clathrin: light polypeptide chain (Lc), heavy polypeptide (Hc); myosin: light polypeptide chain (Lc), heavy polypeptide (Hc); CDC: cell division cycle. UBA1: ubiquitin-activating enzyme E1; UBA2: ubiquitin-like modifier activating enzyme 2; Ufm1: ubiquitin-fold modifier conjugating enzyme 1; UBE: ubiquitin-conjugating enzyme. CCT: chaperonin-containing TCP-1; Psma: proteasome (prosome, macropain) subunit, alpha type; Psmb: proteasome (prosome, macropain) subunit, beta type; Psmc: proteasome (prosome, macropain) 26S ATPase subunit; Psmd: proteasome (prosome, macropain) 26S non-ATPase subunit; TF: transcription factor. MAX: Myc associated factor X; LRR: leucine rich repeat containing protein; LZIC: leucine zipper and CTNNBIP1 domain containing; Bzw1: basic leucine zipper and W2 domain 1. Anp32: acidic (leucine-rich) nuclear phosphoprotein 32.
H/L values are calculated at the average of two independent sample preparations; “-” represents weak or undetected peaks.

Table S8. Sequence Identity of Protein Isoform Pairs

Accession #	Protein name	Mass(kDa)	Amino acids	Identity	Accession #	Protein name	Mass(kDa)	Amino acids	Identity
gi|78000192	TPM1, alpha	32.8	284		gi|85060507	hnRNP, A1	34.8	373	
gi|66730475	TPM2, beta	32.9	284	83.1%	gi|3329498	hnRNP, A2/B1	36.0	341	77.6%
gi|149751320	TPM3, gamma	29.0	248		gi|6754222	hnRNP, A/B	30.9	285	
gi|47894398	TPM4	28.5	248	73.2%	gi|116256514	hnRNP, D	33.8	306	66.5%
gi|3065925	14-3-3, beta	28.1	246		gi|19527048	hnRNP, F	46.5	415	
gi|3065929	14-3-3, gamma	28.4	247	75.0%	gi|10946928	hnRNP, H	49.9	449	74.6%
gi|5803225	14-3-3, epsilon	29.1	255		gi|6754208	Hmg B1	26.3	215	
gi|1526539	14-3-3, zeta	27.8	245	68.6%	gi|6680229	Hmg B2	25.4	210	82.8%
gi|1526541	14-3-3, eta	28.2	246		gi|6680229	Hmg B2	25.4	210	
gi|6756039	14-3-3, theta	27.8	245	71.5%	gi|6680231	Hmg B3	24.3	200	72.7%
gi|1526539	14-3-3, zeta	27.8	245		gi|53733821	tubulin, alpha 1A	51.3	451	
gi|6756039	14-3-3, theta	27.8	245	78.8%	gi|34740335	tubulin, alpha 1B	50.8	451	99.3%
gi|387090	actin, alpha	41.8	375		gi|91855	tubulin, beta 1	51.2	450	
gi|187951999	actin, beta	41.7	375	94.1%	gi|4507729	tubulin, beta 2	50.3	445	80.0%
gi|809561	actin, gamma	41.2	368		gi|13542680	tubulin, beta 2C	50.2	445	
gi|387090	actin, alpha	41.8	375	95.1%	gi|12963615	tubulin, beta 3	50.8	450	92.8%
gi|61097906	actinin, alpha 1	103.1	892		gi|12963615	tubulin, beta 3	50.8	450	
gi|11230802	actinin, alpha 4	105.0	912	86.5%	gi|29145006	tubulin, beta 4	49.6	444	93.2%
gi|435585	myosin, Lc3	15.8	141		gi|7106439	tubulin, beta 5	50.1	444	
gi|33620739	myosin, Lc6	17.0	151	95.0%	gi|27754056	tubulin, beta 6	50.5	447	93.0%
gi|31981605	myosin, Lc4	21.3	193		gi|124517663	annexin A1	39.0	346	
gi|33620739	myosin, Lc6	17.3	151	74.2%	gi|6996913	annexin A2	38.9	339	53.6%
gi|6755714	transgelin 1	22.6	201		gi|148688409	annexin A3	36.6	323	
gi|30519911	transgelin 2	22.6	199	67.5%	gi|161016799	annexin A4	36.8	319	51.1%
gi|122939192	clathrin, Lca	24.0	218		gi|161016799	annexin A4	36.8	319	
gi|30794164	clathrin, Lcb	23.7	211	62.4%	gi|6753060	annexin A5	35.8	319	56.2%
gi|6754976	peroxiredoxin 1	22.9	199		gi|5902076	Sfrs 1	28.1	248	
gi|148747558	peroxiredoxin 2	21.9	198	74.4%	gi|6755478	Sfrs 2	25.8	221	56.9%
gi|148669902	peroxiredoxin 3	28.1	257		gi|119226243	Sfrs 5	31.5	269	
gi|7948999	peroxiredoxin 4	31.3	274	65.0%	gi|15080592	Sfrs 6	39.8	339	64.3%
gi|4507773	UBE2D 1	17.1	147		gi|5689158	septin 6	49.2	429	
gi|4507777	UBE2D 3	17.1	147	88.4%	gi|148693353	septin 7	44.9	384	43.8%
gi|1374782	Nedd4	111.8	957		gi|18700032	Anp32b	31.6	272	
gi|51895883	Nedd4l	103.2	875	64.3%	gi|254587996	Anp32e	30.3	260	61.4%

Sequence alignment using an on-line web tool by BLAST at http://blast.ncbi.nlm.nih.gov/Blast.cgi
