# Supplementary material for: Quantification of protein isoforms in mesenchymal stem cells by reductive dimethylation of lysines in intact proteins
Source: Proteomics. 2012 Jan 13;12(3):369–79. doi: 10.1002/pmic.201100308 (PMC3440571; doi:10.1002/pmic.201100308)
Supplement: Supplementary file 2 [file pmic0012-0369-SD2.ppt]

## Slide 1
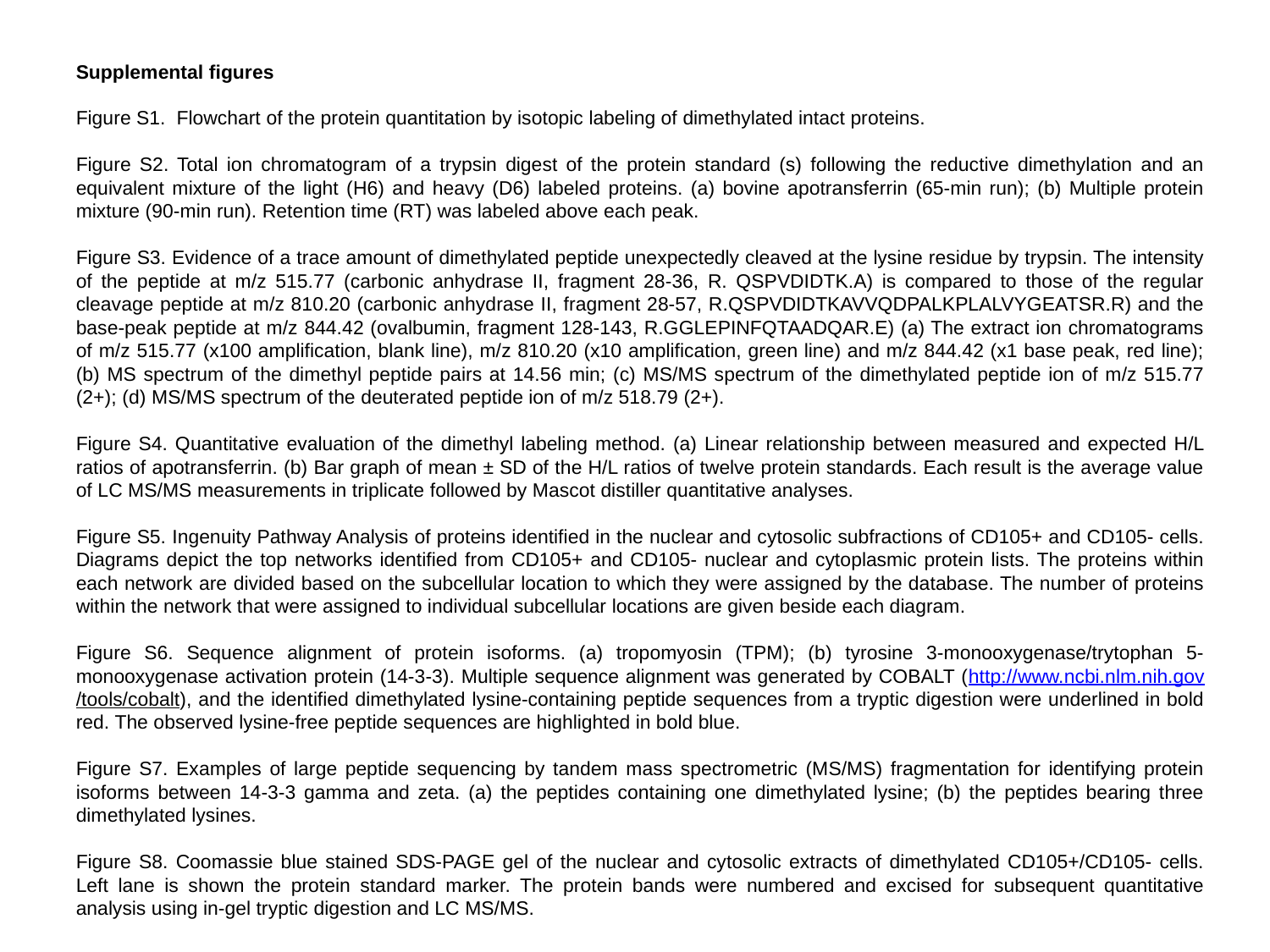

Supplemental figures
Figure S1. Flowchart of the protein quantitation by isotopic labeling of dimethylated intact proteins.
Figure S2. Total ion chromatogram of a trypsin digest of the protein standard (s) following the reductive dimethylation and an equivalent mixture of the light (H6) and heavy (D6) labeled proteins. (a) bovine apotransferrin (65-min run); (b) Multiple protein mixture (90-min run). Retention time (RT) was labeled above each peak.
Figure S3. Evidence of a trace amount of dimethylated peptide unexpectedly cleaved at the lysine residue by trypsin. The intensity of the peptide at m/z 515.77 (carbonic anhydrase II, fragment 28-36, R. QSPVDIDTK.A) is compared to those of the regular cleavage peptide at m/z 810.20 (carbonic anhydrase II, fragment 28-57, R.QSPVDIDTKAVVQDPALKPLALVYGEATSR.R) and the base-peak peptide at m/z 844.42 (ovalbumin, fragment 128-143, R.GGLEPINFQTAADQAR.E) (a) The extract ion chromatograms of m/z 515.77 (x100 amplification, blank line), m/z 810.20 (x10 amplification, green line) and m/z 844.42 (x1 base peak, red line); (b) MS spectrum of the dimethyl peptide pairs at 14.56 min; (c) MS/MS spectrum of the dimethylated peptide ion of m/z 515.77 (2+); (d) MS/MS spectrum of the deuterated peptide ion of m/z 518.79 (2+).
Figure S4. Quantitative evaluation of the dimethyl labeling method. (a) Linear relationship between measured and expected H/L ratios of apotransferrin. (b) Bar graph of mean ± SD of the H/L ratios of twelve protein standards. Each result is the average value of LC MS/MS measurements in triplicate followed by Mascot distiller quantitative analyses.
Figure S5. Ingenuity Pathway Analysis of proteins identified in the nuclear and cytosolic subfractions of CD105+ and CD105- cells. Diagrams depict the top networks identified from CD105+ and CD105- nuclear and cytoplasmic protein lists. The proteins within each network are divided based on the subcellular location to which they were assigned by the database. The number of proteins within the network that were assigned to individual subcellular locations are given beside each diagram.
Figure S6. Sequence alignment of protein isoforms. (a) tropomyosin (TPM); (b) tyrosine 3-monooxygenase/trytophan 5-monooxygenase activation protein (14-3-3). Multiple sequence alignment was generated by COBALT (http://www.ncbi.nlm.nih.gov/tools/cobalt), and the identified dimethylated lysine-containing peptide sequences from a tryptic digestion were underlined in bold red. The observed lysine-free peptide sequences are highlighted in bold blue.
Figure S7. Examples of large peptide sequencing by tandem mass spectrometric (MS/MS) fragmentation for identifying protein isoforms between 14-3-3 gamma and zeta. (a) the peptides containing one dimethylated lysine; (b) the peptides bearing three dimethylated lysines.
Figure S8. Coomassie blue stained SDS-PAGE gel of the nuclear and cytosolic extracts of dimethylated CD105+/CD105- cells. Left lane is shown the protein standard marker. The protein bands were numbered and excised for subsequent quantitative analysis using in-gel tryptic digestion and LC MS/MS.

## Slide 2
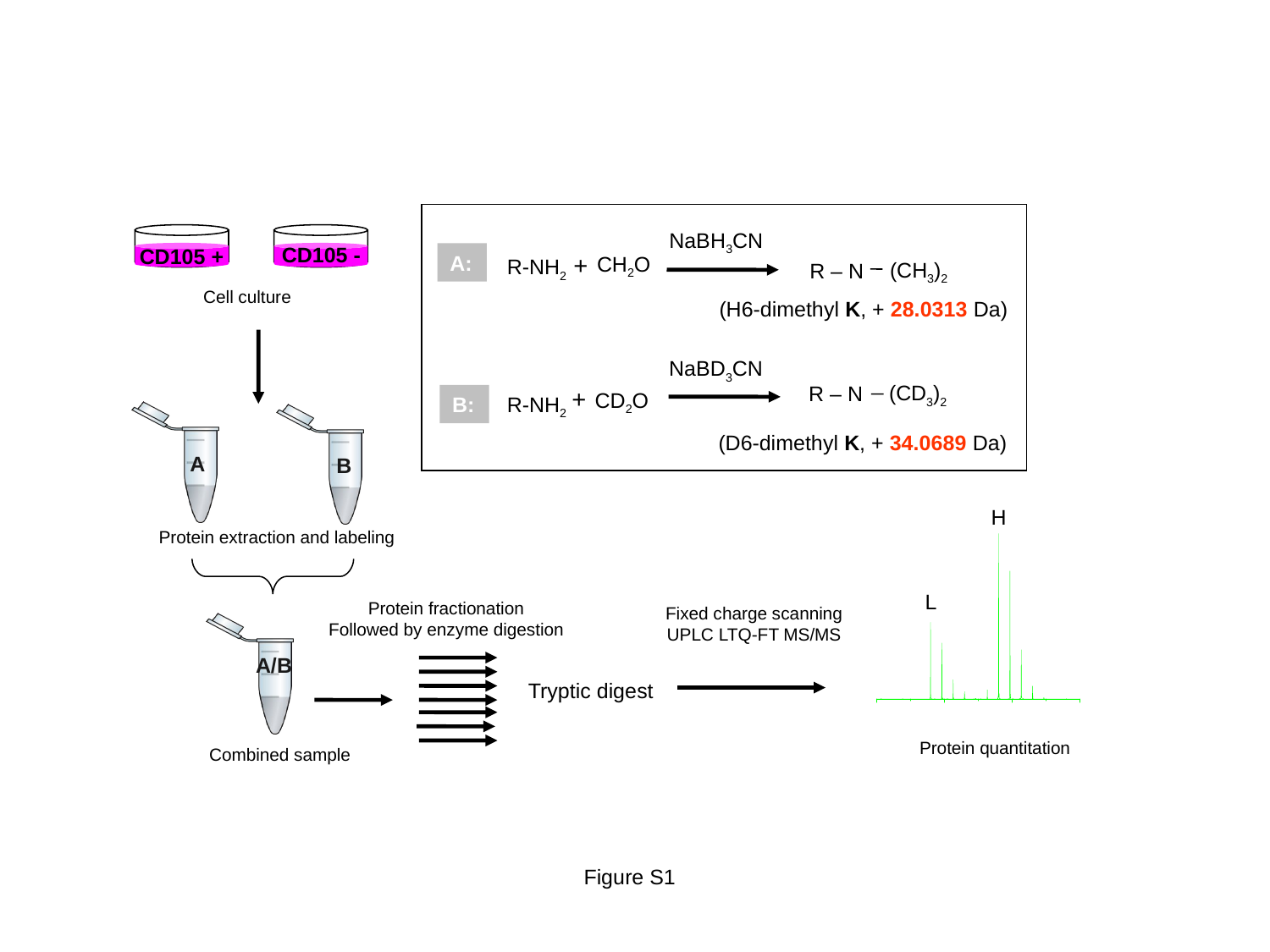

NaBH3CN
CD105 -
CD105 +
A:
+
CH2O
R-NH2
(CH3)2
R – N
Cell culture
(H6-dimethyl K, + 28.0313 Da)
NaBD3CN
(CD3)2
R – N
+
CD2O
B:
R-NH2
(D6-dimethyl K, + 34.0689 Da)
A
B
H
Protein extraction and labeling
L
Protein fractionation
Followed by enzyme digestion
Fixed charge scanning
UPLC LTQ-FT MS/MS
A/B
Tryptic digest
Protein quantitation
Combined sample
Figure S1

## Slide 3
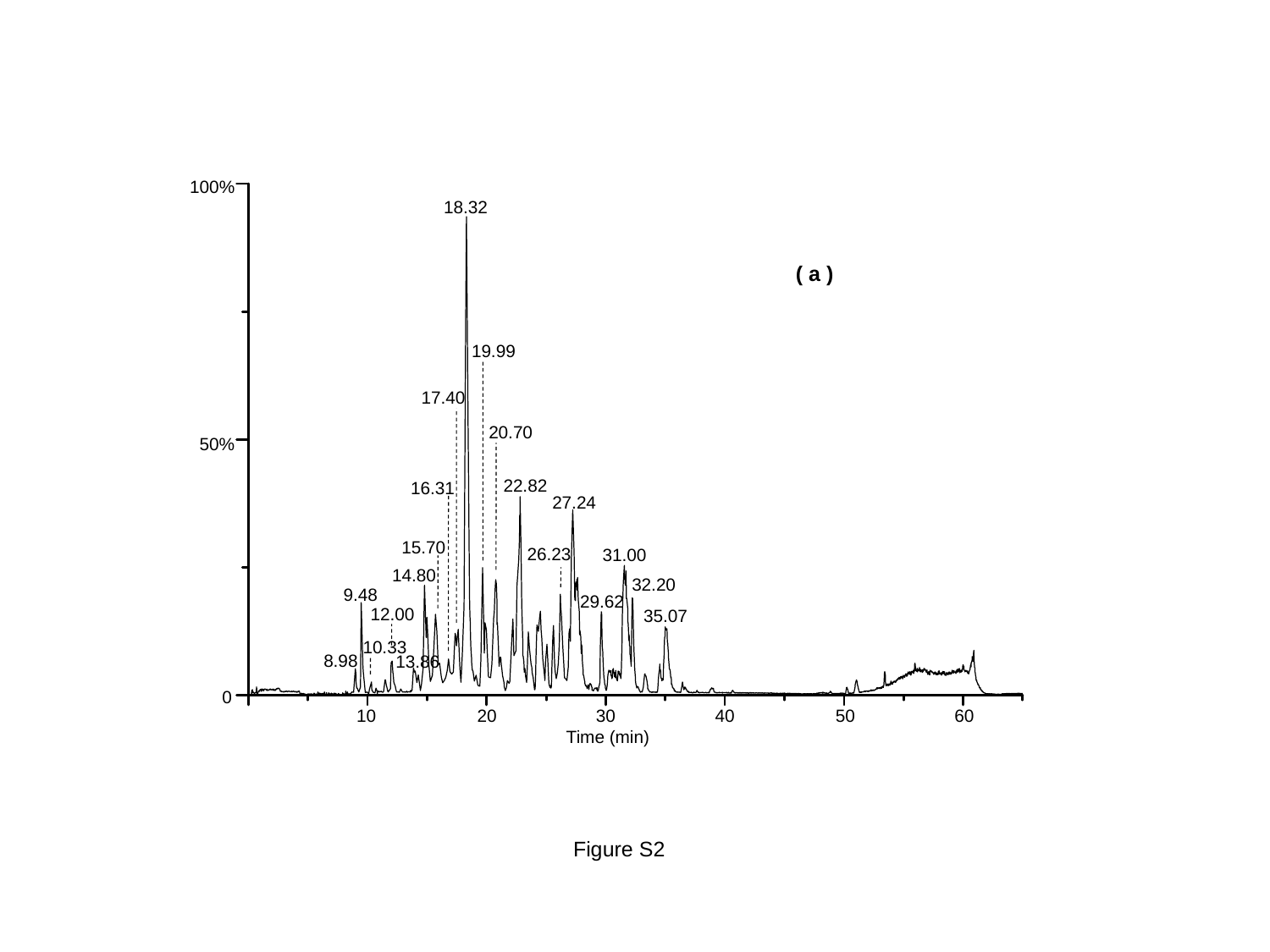

100%
18.32
( a )
19.99
17.40
20.70
50%
22.82
16.31
27.24
15.70
26.23
31.00
14.80
32.20
9.48
29.62
12.00
35.07
10.33
8.98
13.86
0
10
20
30
40
50
60
Time (min)
Figure S2

## Slide 4
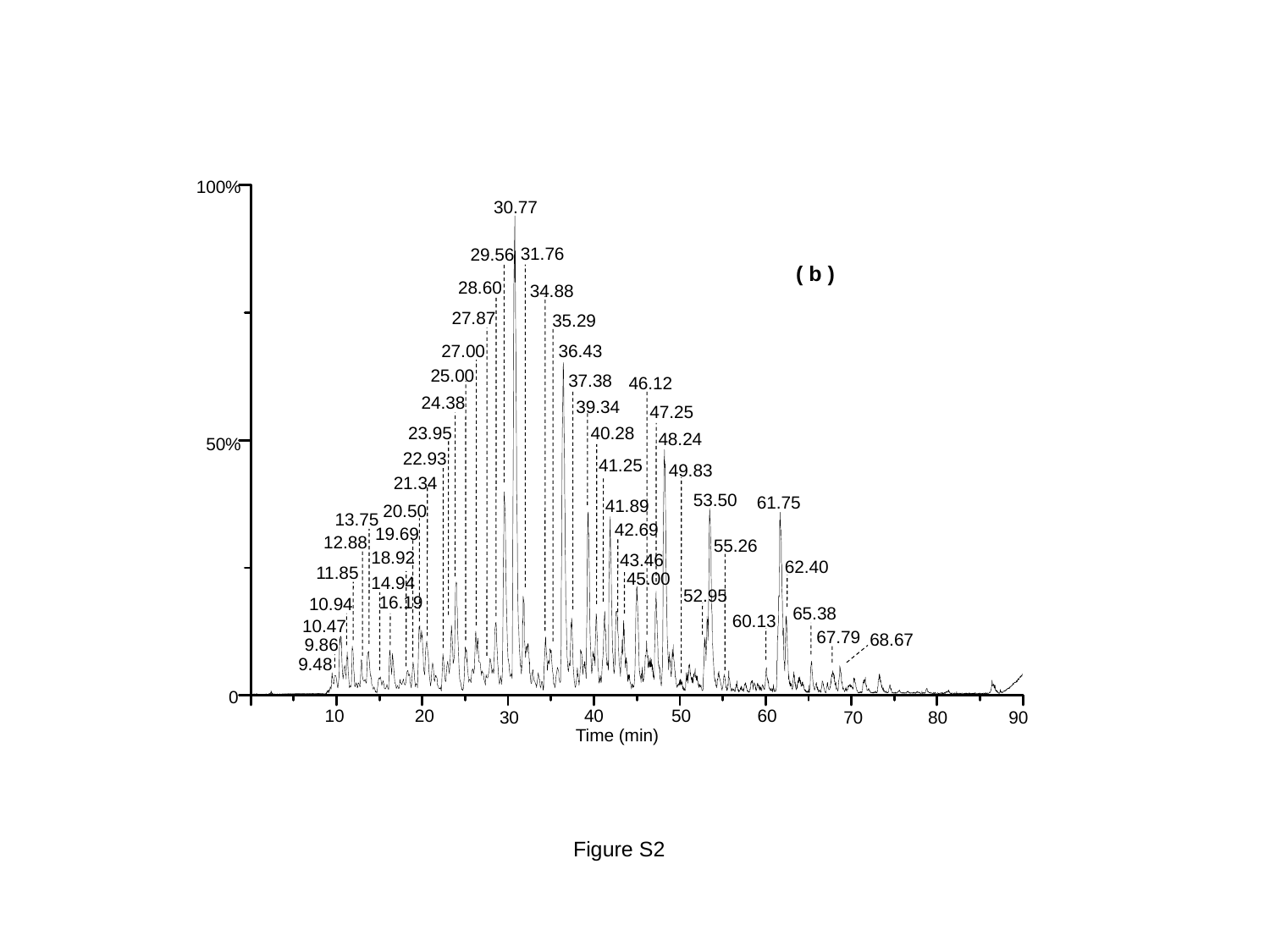

100%
30.77
31.76
29.56
( b )
28.60
34.88
27.87
35.29
36.43
27.00
25.00
37.38
46.12
24.38
39.34
47.25
40.28
23.95
48.24
50%
22.93
41.25
49.83
21.34
53.50
61.75
41.89
20.50
13.75
42.69
19.69
12.88
55.26
18.92
43.46
62.40
11.85
45.00
14.94
52.95
16.19
10.94
65.38
60.13
10.47
67.79
68.67
9.86
9.48
0
10
20
40
50
60
30
70
80
90
Time (min)
Figure S2

## Slide 5
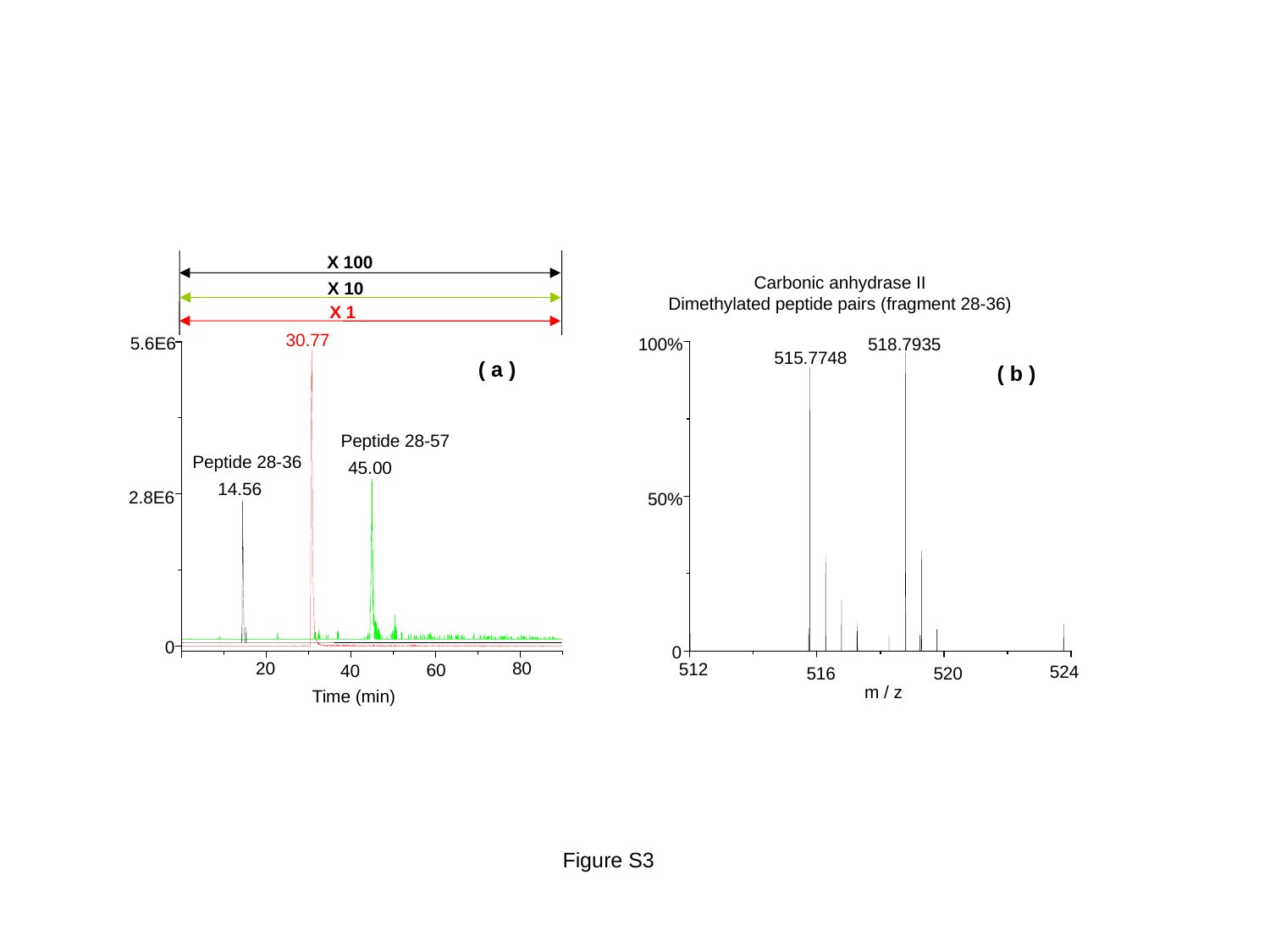

X 100
Carbonic anhydrase II
Dimethylated peptide pairs (fragment 28-36)
X 10
X 1
30.77
5.6E6
518.7935
100%
515.7748
( a )
( b )
Peptide 28-57
Peptide 28-36
45.00
14.56
2.8E6
50%
0
0
20
80
512
60
40
524
516
520
m / z
Time (min)
Figure S3

## Slide 6
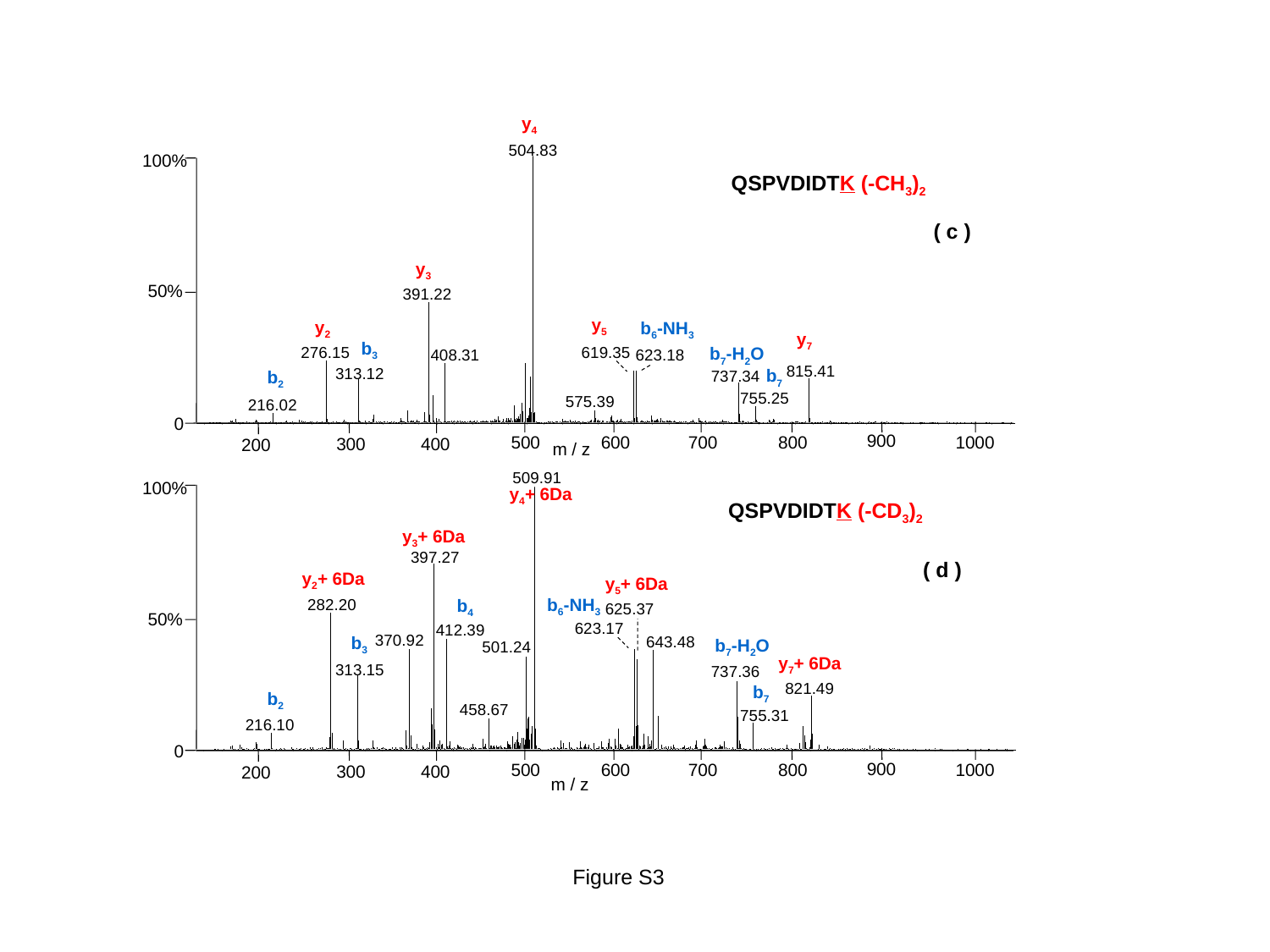

y4
504.83
100%
QSPVDIDTK (-CH3)2
( c )
y3
50%
391.22
y5
y2
b6-NH3
y7
b3
276.15
619.35
b7-H2O
408.31
623.18
815.41
313.12
b7
b2
737.34
755.25
575.39
216.02
0
900
500
600
700
800
1000
300
400
200
m / z
509.91
100%
y4+ 6Da
QSPVDIDTK (-CD3)2
y3+ 6Da
397.27
( d )
y2+ 6Da
y5+ 6Da
b6-NH3
282.20
b4
625.37
50%
623.17
412.39
370.92
b3
643.48
b7-H2O
501.24
y7+ 6Da
313.15
737.36
821.49
b7
b2
458.67
755.31
216.10
0
900
500
600
700
800
1000
300
400
200
m / z
Figure S3

## Slide 7
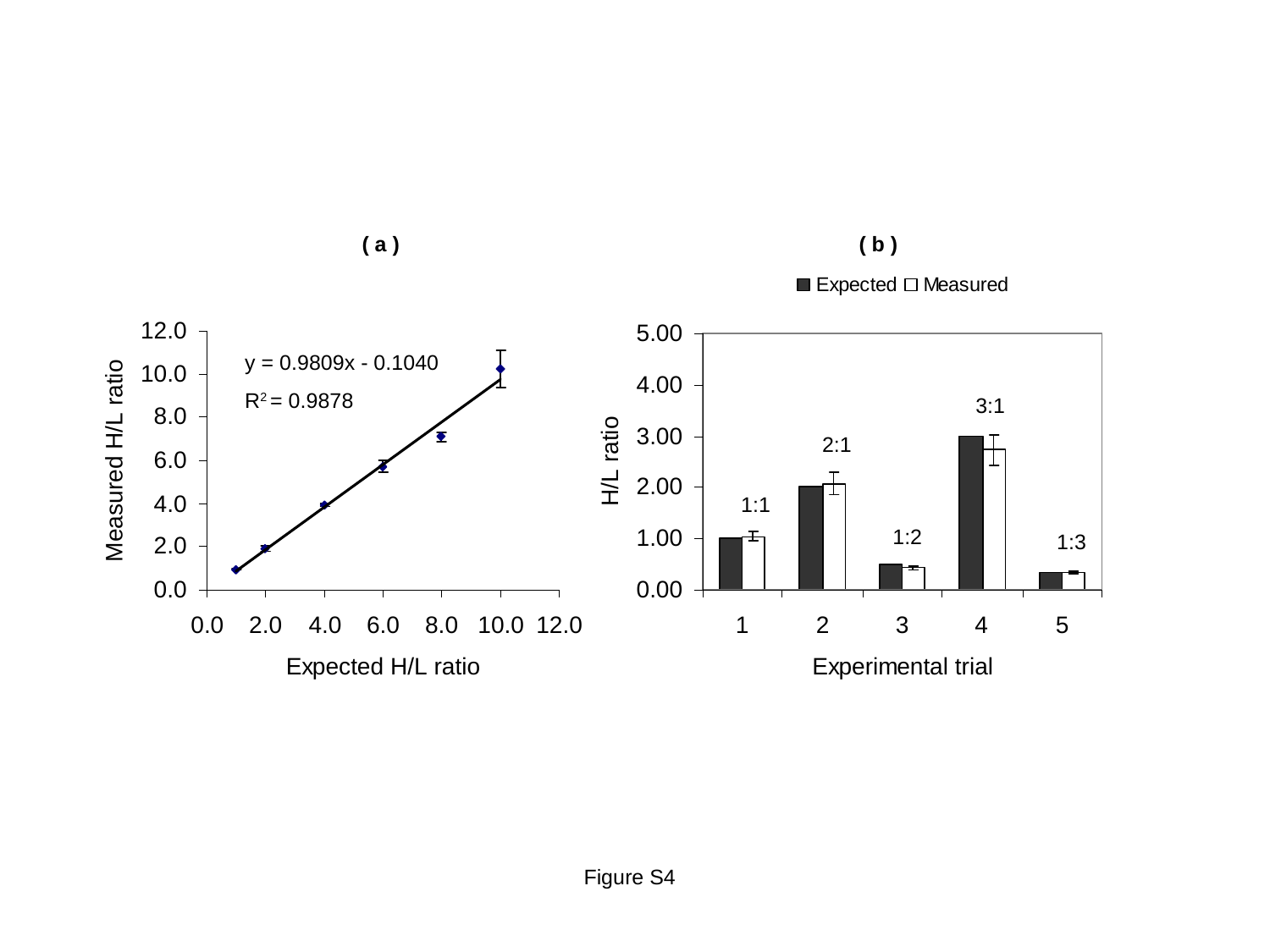

( b )
( a )
y = 0.9809x - 0.1040
R2 = 0.9878
3:1
2:1
1:1
1:2
1:3
Figure S4

## Slide 8
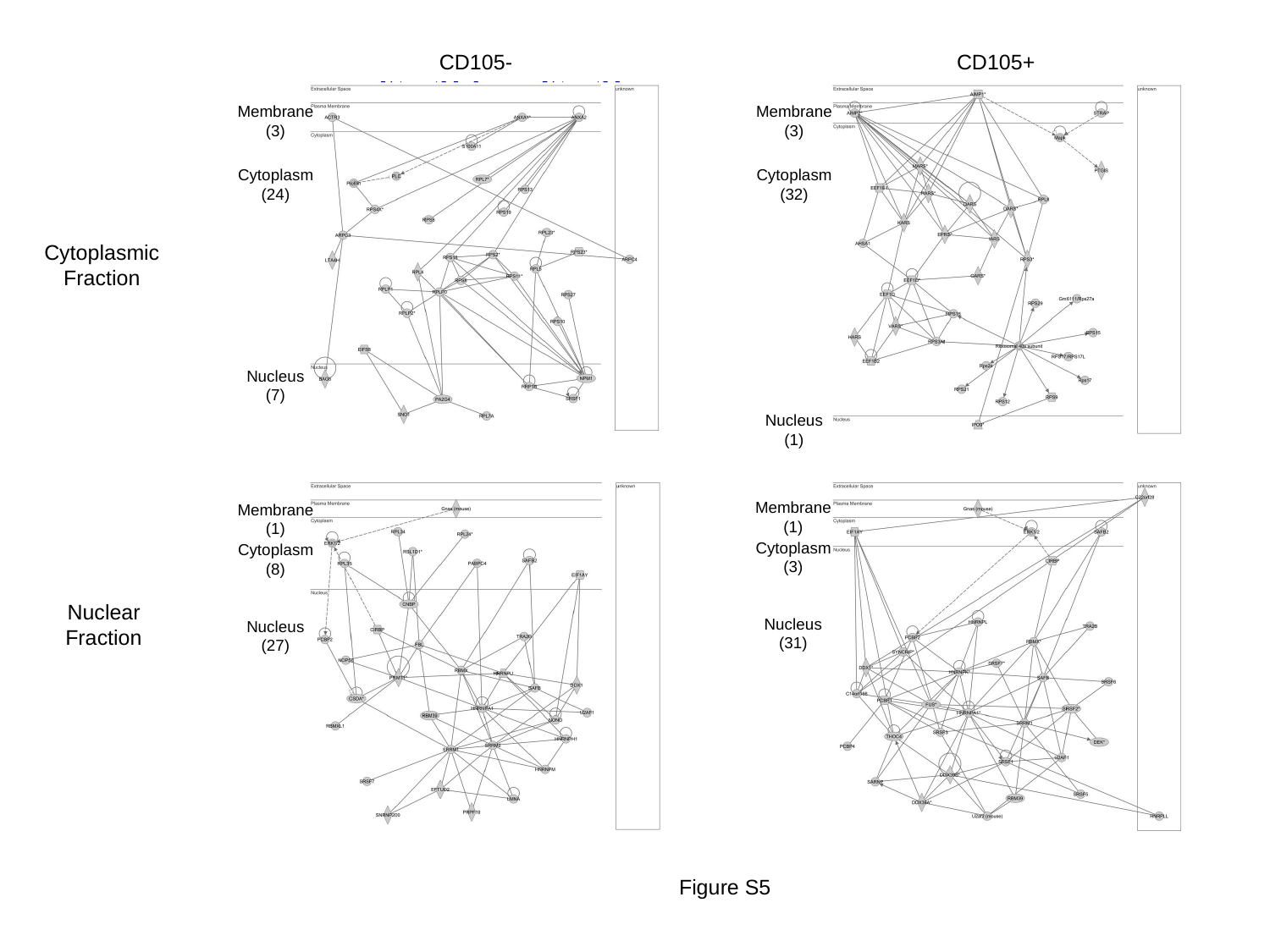

CD105-
CD105+
Membrane
(3)
Membrane
(3)
Cytoplasm
(24)
Cytoplasm
(32)
Cytoplasmic
Fraction
Nucleus
(7)
Nucleus
(1)
Membrane
(1)
Membrane
(1)
Cytoplasm
(3)
Cytoplasm
(8)
Nuclear
Fraction
Nucleus
(31)
Nucleus
(27)
Figure S5

## Slide 9
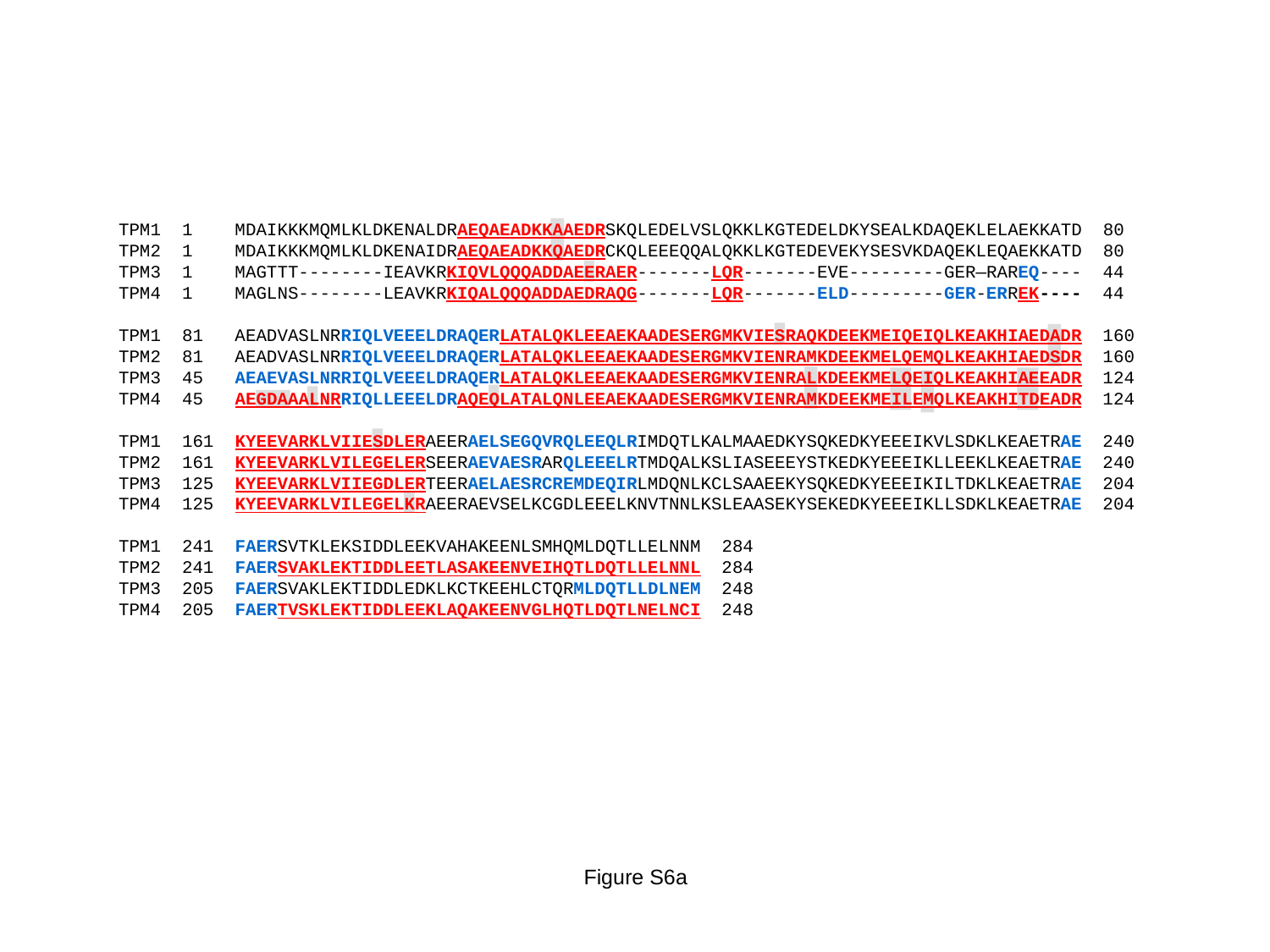

TPM1 1 MDAIKKKMQMLKLDKENALDRAEQAEADKKAAEDRSKQLEDELVSLQKKLKGTEDELDKYSEALKDAQEKLELAEKKATD 80
TPM2 1 MDAIKKKMQMLKLDKENAIDRAEQAEADKKQAEDRCKQLEEEQQALQKKLKGTEDEVEKYSESVKDAQEKLEQAEKKATD 80
TPM3 1 MAGTTT--------IEAVKRKIQVLQQQADDAEERAER-------LQR-------EVE---------GER—RAREQ---- 44
TPM4 1 MAGLNS--------LEAVKRKIQALQQQADDAEDRAQG-------LQR-------ELD---------GER-ERREK---- 44
TPM1 81 AEADVASLNRRIQLVEEELDRAQERLATALQKLEEAEKAADESERGMKVIESRAQKDEEKMEIQEIQLKEAKHIAEDADR 160
TPM2 81 AEADVASLNRRIQLVEEELDRAQERLATALQKLEEAEKAADESERGMKVIENRAMKDEEKMELQEMQLKEAKHIAEDSDR 160
TPM3 45 AEAEVASLNRRIQLVEEELDRAQERLATALQKLEEAEKAADESERGMKVIENRALKDEEKMELQEIQLKEAKHIAEEADR 124
TPM4 45 AEGDAAALNRRIQLLEEELDRAQEQLATALQNLEEAEKAADESERGMKVIENRAMKDEEKMEILEMQLKEAKHITDEADR 124
TPM1 161 KYEEVARKLVIIESDLERAEERAELSEGQVRQLEEQLRIMDQTLKALMAAEDKYSQKEDKYEEEIKVLSDKLKEAETRAE 240
TPM2 161 KYEEVARKLVILEGELERSEERAEVAESRARQLEEELRTMDQALKSLIASEEEYSTKEDKYEEEIKLLEEKLKEAETRAE 240
TPM3 125 KYEEVARKLVIIEGDLERTEERAELAESRCREMDEQIRLMDQNLKCLSAAEEKYSQKEDKYEEEIKILTDKLKEAETRAE 204
TPM4 125 KYEEVARKLVILEGELKRAEERAEVSELKCGDLEEELKNVTNNLKSLEAASEKYSEKEDKYEEEIKLLSDKLKEAETRAE 204
TPM1 241 FAERSVTKLEKSIDDLEEKVAHAKEENLSMHQMLDQTLLELNNM 284
TPM2 241 FAERSVAKLEKTIDDLEETLASAKEENVEIHQTLDQTLLELNNL 284
TPM3 205 FAERSVAKLEKTIDDLEDKLKCTKEEHLCTQRMLDQTLLDLNEM 248
TPM4 205 FAERTVSKLEKTIDDLEEKLAQAKEENVGLHQTLDQTLNELNCI 248
Figure S6a

## Slide 10
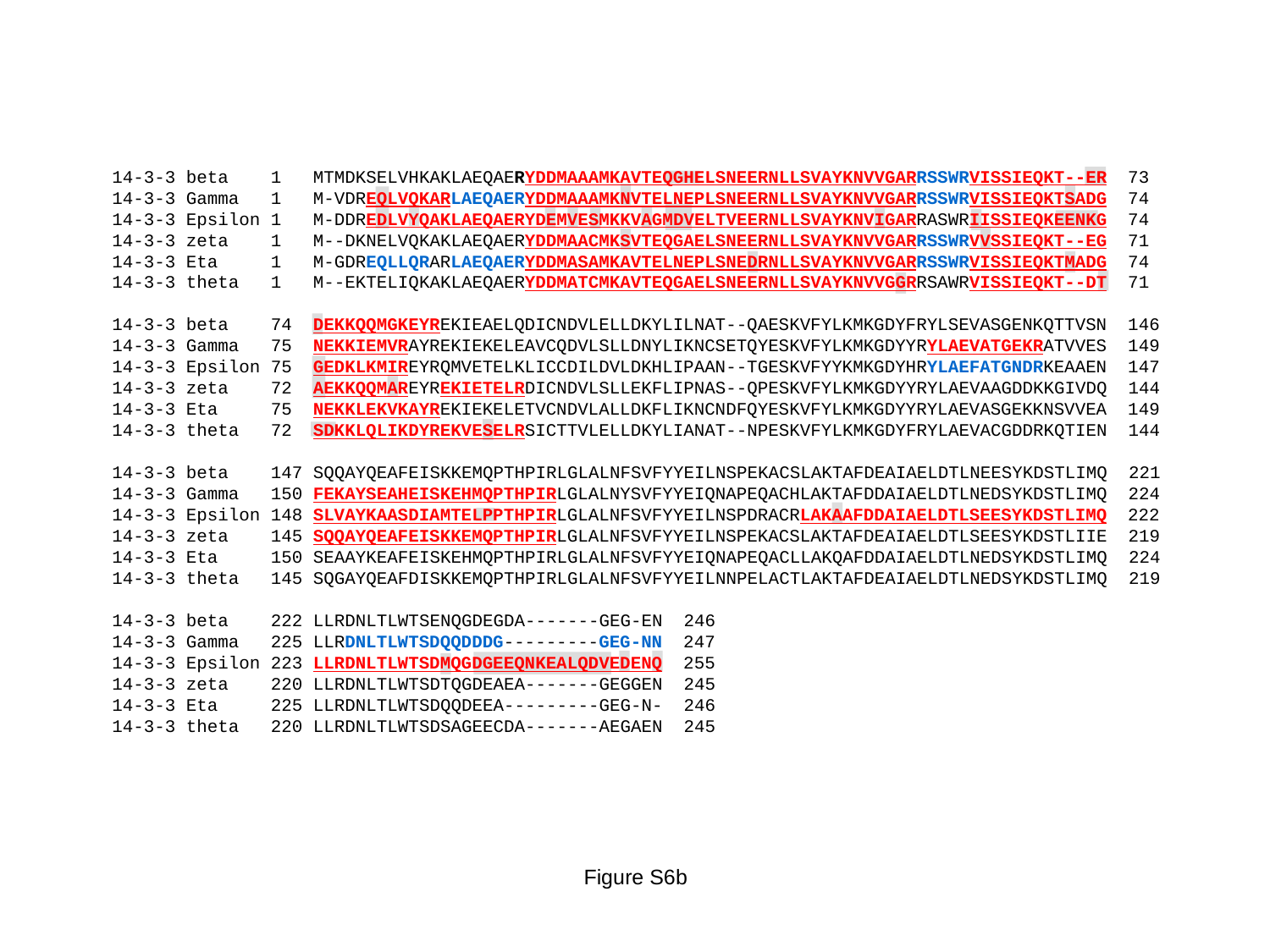

14-3-3 beta 1 MTMDKSELVHKAKLAEQAERYDDMAAAMKAVTEQGHELSNEERNLLSVAYKNVVGARRSSWRVISSIEQKT--ER 73
14-3-3 Gamma 1 M-VDREQLVQKARLAEQAERYDDMAAAMKNVTELNEPLSNEERNLLSVAYKNVVGARRSSWRVISSIEQKTSADG 74
14-3-3 Epsilon 1 M-DDREDLVYQAKLAEQAERYDEMVESMKKVAGMDVELTVEERNLLSVAYKNVIGARRASWRIISSIEQKEENKG 74
14-3-3 zeta 1 M--DKNELVQKAKLAEQAERYDDMAACMKSVTEQGAELSNEERNLLSVAYKNVVGARRSSWRVVSSIEQKT--EG 71
14-3-3 Eta 1 M-GDREQLLQRARLAEQAERYDDMASAMKAVTELNEPLSNEDRNLLSVAYKNVVGARRSSWRVISSIEQKTMADG 74
14-3-3 theta 1 M--EKTELIQKAKLAEQAERYDDMATCMKAVTEQGAELSNEERNLLSVAYKNVVGGRRSAWRVISSIEQKT--DT 71
14-3-3 beta 74 DEKKQQMGKEYREKIEAELQDICNDVLELLDKYLILNAT--QAESKVFYLKMKGDYFRYLSEVASGENKQTTVSN 146
14-3-3 Gamma 75 NEKKIEMVRAYREKIEKELEAVCQDVLSLLDNYLIKNCSETQYESKVFYLKMKGDYYRYLAEVATGEKRATVVES 149
14-3-3 Epsilon 75 GEDKLKMIREYRQMVETELKLICCDILDVLDKHLIPAAN--TGESKVFYYKMKGDYHRYLAEFATGNDRKEAAEN 147
14-3-3 zeta 72 AEKKQQMAREYREKIETELRDICNDVLSLLEKFLIPNAS--QPESKVFYLKMKGDYYRYLAEVAAGDDKKGIVDQ 144
14-3-3 Eta 75 NEKKLEKVKAYREKIEKELETVCNDVLALLDKFLIKNCNDFQYESKVFYLKMKGDYYRYLAEVASGEKKNSVVEA 149
14-3-3 theta 72 SDKKLQLIKDYREKVESELRSICTTVLELLDKYLIANAT--NPESKVFYLKMKGDYFRYLAEVACGDDRKQTIEN 144
14-3-3 beta 147 SQQAYQEAFEISKKEMQPTHPIRLGLALNFSVFYYEILNSPEKACSLAKTAFDEAIAELDTLNEESYKDSTLIMQ 221
14-3-3 Gamma 150 FEKAYSEAHEISKEHMQPTHPIRLGLALNYSVFYYEIQNAPEQACHLAKTAFDDAIAELDTLNEDSYKDSTLIMQ 224
14-3-3 Epsilon 148 SLVAYKAASDIAMTELPPTHPIRLGLALNFSVFYYEILNSPDRACRLAKAAFDDAIAELDTLSEESYKDSTLIMQ 222
14-3-3 zeta 145 SQQAYQEAFEISKKEMQPTHPIRLGLALNFSVFYYEILNSPEKACSLAKTAFDEAIAELDTLSEESYKDSTLIIE 219
14-3-3 Eta 150 SEAAYKEAFEISKEHMQPTHPIRLGLALNFSVFYYEIQNAPEQACLLAKQAFDDAIAELDTLNEDSYKDSTLIMQ 224
14-3-3 theta 145 SQGAYQEAFDISKKEMQPTHPIRLGLALNFSVFYYEILNNPELACTLAKTAFDEAIAELDTLNEDSYKDSTLIMQ 219
14-3-3 beta 222 LLRDNLTLWTSENQGDEGDA-------GEG-EN 246
14-3-3 Gamma 225 LLRDNLTLWTSDQQDDDG---------GEG-NN 247
14-3-3 Epsilon 223 LLRDNLTLWTSDMQGDGEEQNKEALQDVEDENQ 255
14-3-3 zeta 220 LLRDNLTLWTSDTQGDEAEA-------GEGGEN 245
14-3-3 Eta 225 LLRDNLTLWTSDQQDEEA---------GEG-N- 246
14-3-3 theta 220 LLRDNLTLWTSDSAGEECDA-------AEGAEN 245
Figure S6b

## Slide 11
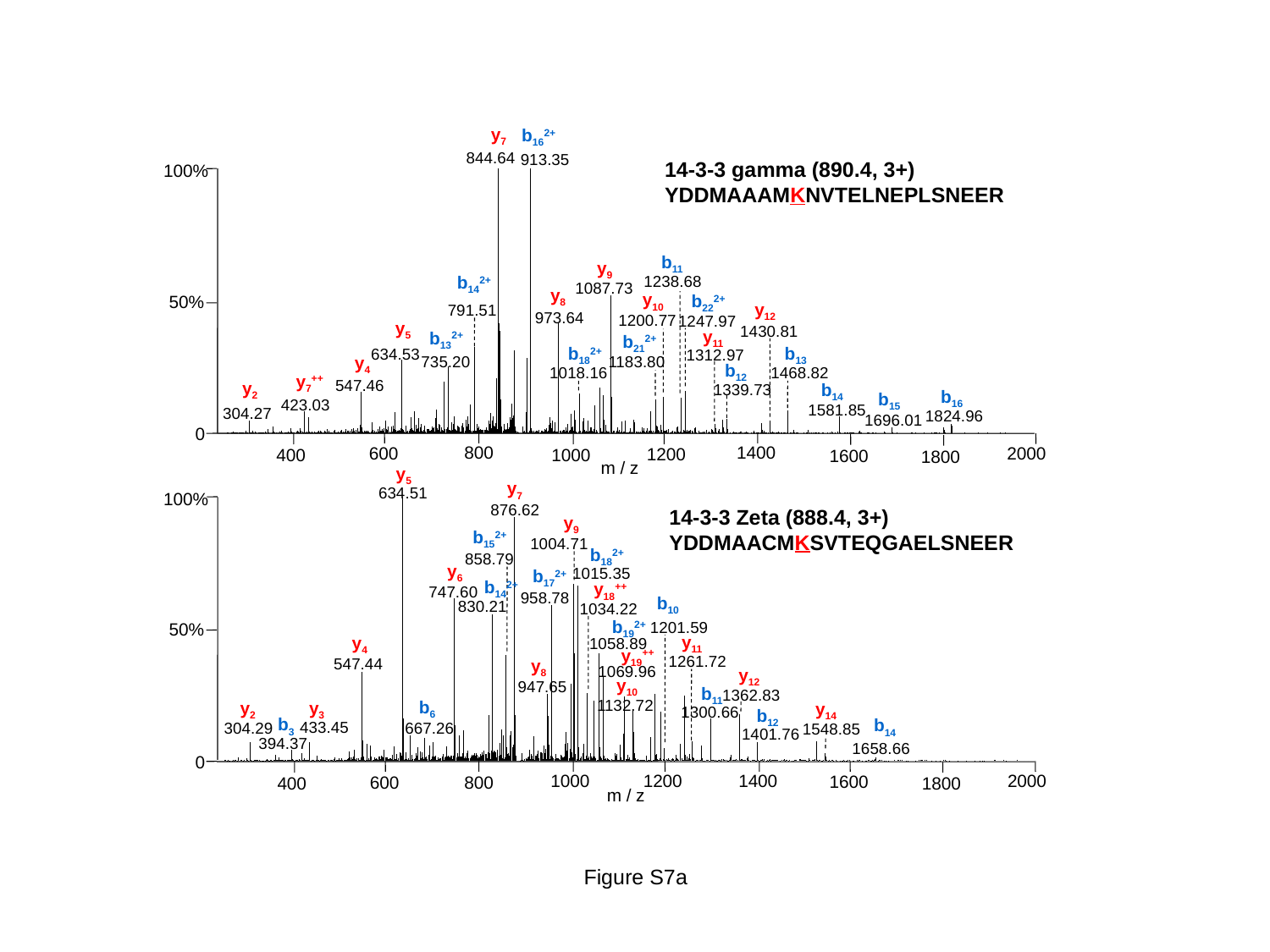

y7
b162+
844.64
913.35
14-3-3 gamma (890.4, 3+)
YDDMAAAMKNVTELNEPLSNEER
100%
b11
y9
b142+
1238.68
1087.73
y8
y10
b222+
50%
y12
791.51
973.64
1200.77
1247.97
y5
1430.81
y11
b132+
b212+
b182+
b13
634.53
1312.97
y4
735.20
1183.80
b12
1018.16
1468.82
y7++
547.46
y2
b14
1339.73
b16
b15
423.03
1581.85
304.27
1824.96
1696.01
0
800
1400
600
2000
1200
400
1000
1600
1800
m / z
y5
y7
634.51
100%
876.62
14-3-3 Zeta (888.4, 3+)
YDDMAACMKSVTEQGAELSNEER
y9
b152+
1004.71
b182+
858.79
y6
1015.35
b172+
b142+
y18++
747.60
958.78
b10
830.21
1034.22
b192+
1201.59
50%
y11
y4
1058.89
y19++
1261.72
547.44
y8
1069.96
y12
y10
947.65
b11
1362.83
1132.72
b6
y3
y2
y14
1300.66
b12
b3
b14
433.45
304.29
 667.26
1548.85
1401.76
394.37
1658.66
0
1000
1200
1400
2000
1600
600
800
1800
400
m / z
Figure S7a

## Slide 12
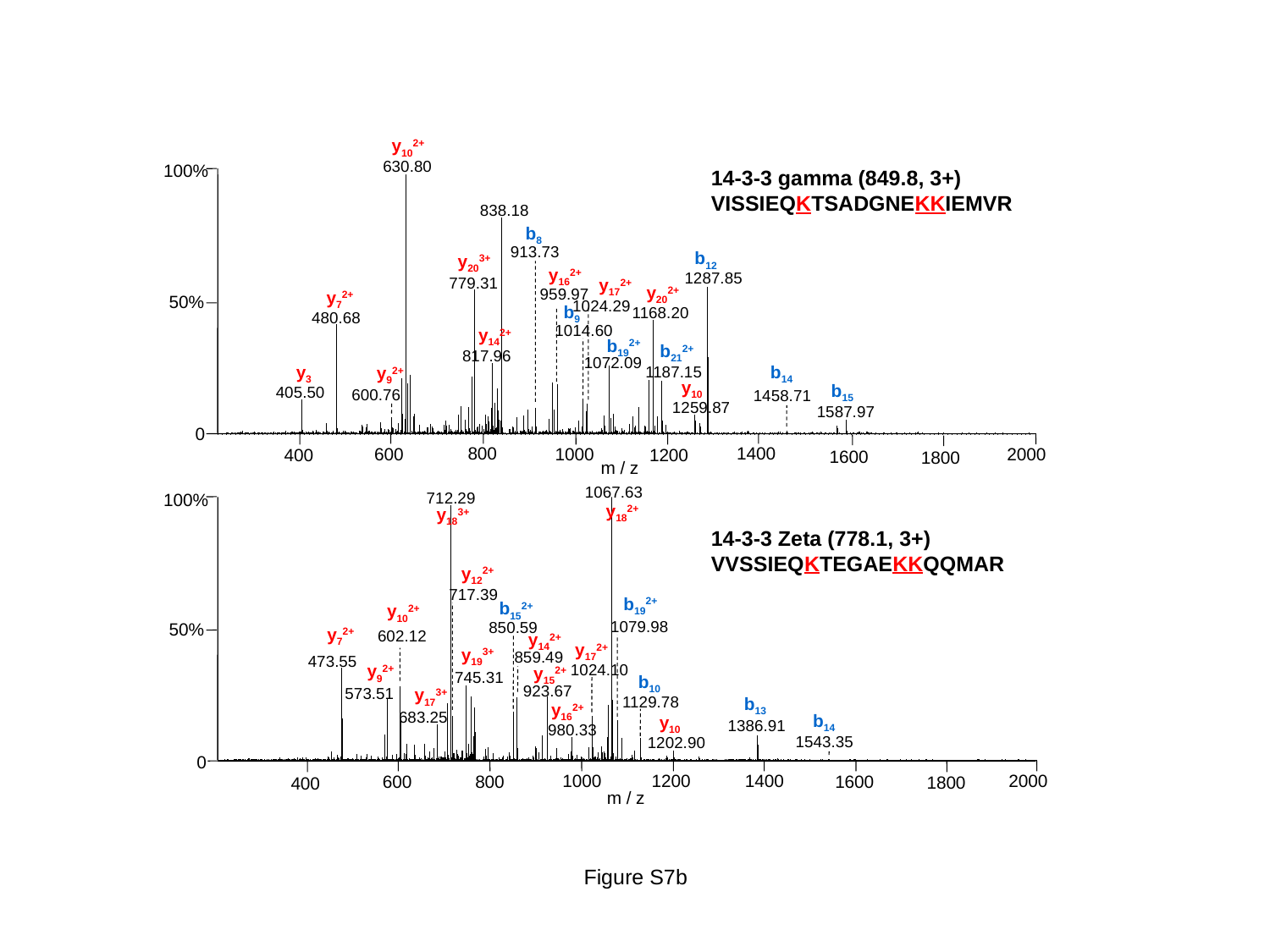

100%
50%
0
800
1400
600
2000
1000
1200
400
1600
1800
m / z
100%
50%
0
1000
1200
1400
2000
600
1600
800
1800
400
m / z
y102+
630.80
14-3-3 gamma (849.8, 3+)
VISSIEQKTSADGNEKKIEMVR
838.18
b8
913.73
b12
y203+
y162+
1287.85
779.31
y172+
y202+
959.97
y72+
1024.29
b9
1168.20
480.68
1014.60
y142+
b192+
b212+
817.96
1072.09
b14
y3
1187.15
y92+
y10
b15
405.50
600.76
1458.71
1259.87
1587.97
1067.63
712.29
y182+
y183+
14-3-3 Zeta (778.1, 3+)
VVSSIEQKTEGAEKKQQMAR
y122+
717.39
b192+
b152+
y102+
1079.98
850.59
y72+
602.12
y142+
y172+
y193+
859.49
473.55
1024.10
y92+
y152+
745.31
b10
923.67
573.51
y173+
1129.78
b13
y162+
683.25
b14
y10
1386.91
980.33
1543.35
1202.90
Figure S7b

## Slide 13
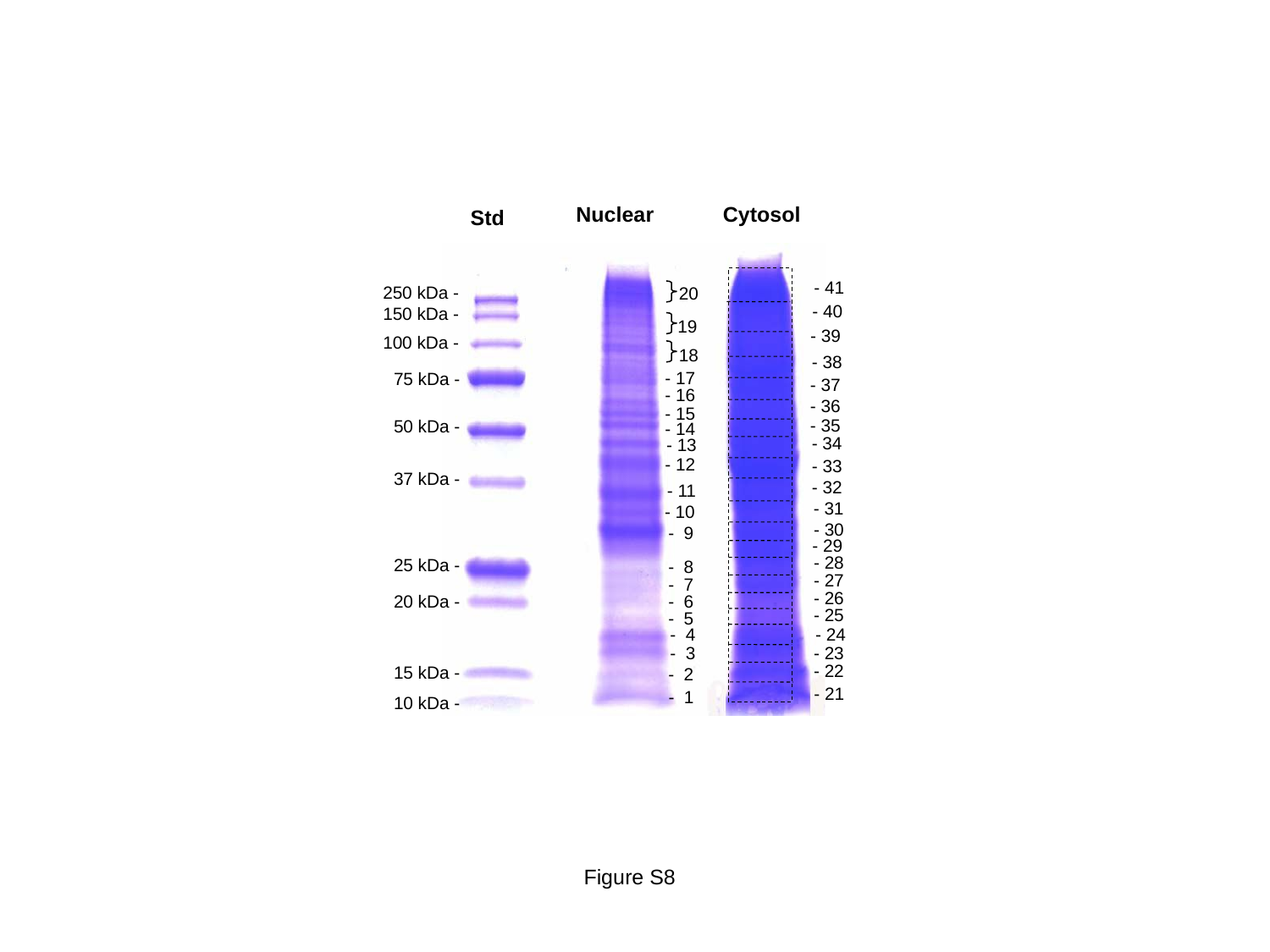

Nuclear
Cytosol
Std
- 41
250 kDa -
20
- 40
150 kDa -
19
- 39
100 kDa -
18
- 38
- 17
75 kDa -
- 37
- 16
- 36
- 15
- 35
50 kDa -
- 14
- 34
- 13
- 12
- 33
37 kDa -
- 32
- 11
- 31
- 10
- 30
- 9
- 29
- 28
25 kDa -
- 8
- 27
- 7
- 26
20 kDa -
- 6
- 25
- 5
- 4
- 24
- 3
- 23
- 22
15 kDa -
- 2
- 21
- 1
10 kDa -
Figure S8
